# NBR1-Mediated Autophagic Degradation of YTHDF1 Curtails *FDX1* Translation to Drive Concurrent Multikinase Inhibitor Resistance and Cuproptosis Tolerance

**DOI:** 10.34133/cancomm.0048

**Published:** 2026-09-11

**Authors:** Weikai Wang, Xin Jiang, Xiaona Chen, Kaiyu Liang, Miaoqin Chen, Kaizhong Lu, Qi Wei, Yuchen Wu, Liyuan Zhu, Yuan Chen, Tianlun Hou, Jingfeng Luo, Chentao Li, Xian Wang, Hongchuan Jin, Lifeng Feng

**Affiliations:** ^1^ Department of Medical Oncology, Zhejiang Key Laboratory of Multi-omics Precision Diagnosis and Treatment of Liver Diseases, Sir Run Run Shaw Hospital, School of Medicine, Zhejiang University, Hangzhou, Zhejiang, P. R. China.; ^2^ Department of Orthopaedic Surgery, Sir Run Run Shaw Hospital, School of Medicine, Zhejiang University, Hangzhou, Zhejiang, P. R. China.

## Abstract

**Background:** Cancer cells frequently acquire adaptive resistance to targeted therapies; however, strategies capable of concurrently overcoming treatment tolerance and reactivating cell death pathways are currently lacking. Here, we investigated the dual role of ferredoxin 1 (FDX1) in modulating both multikinase inhibitor (MKI) sensitivity and cuproptosis susceptibility in hepatocellular carcinoma (HCC), and sought to develop a therapeutic approach for reversing resistance. **Methods:** HCC models, both in vitro and in vivo, were employed to investigate the role of FDX1 in MKI resistance and cuproptosis evasion. Polysome profiling, SunTag translation reporters, CRISPR-Cas9 mutagenesis, and mass spectrometry were employed to delineate the underlying mechanisms. A codelivery nanoliposome system was engineered and tested in orthotopic HCC models. **Results:** Prolonged exposure to MKIs led to the down-regulation of FDX1 protein levels, resulting in MKI resistance and cuproptosis tolerance in HCC both in vitro and in vivo. Mechanistically, we found that MKIs inactivated protein kinase B (PKB, also known as AKT)-mechanistic target of rapamycin (mTOR) signaling, thereby suppressing the SET and MYND domain-containing protein 2 (SMYD2)-mediated methylation of YTH domain family protein 1 (YTHDF1) at lysine 515 (K515). Hypomethylated YTHDF1 was degraded via next to BRCA1 gene 1 protein (NBR1)-dependent autophagy, leading to the repression of N6-methyladenosine modification-dependent translation of *FDX1* mRNA. FDX1 deficiency drove MKI resistance by reactivating AKT survival signaling while impairing cuproptosis through reduced divalent copper ions (Cu^2+^) to monovalent copper ions (Cu^+^) conversion and the loss of protein lipoylation. Additionally, restoring FDX1 expression through *NBR1* knockdown or *YTHDF1* overexpression overcame MKI resistance and resensitized HCC cells to cuproptosis. Finally, a nanoliposomal system, super cuproptosis detonator liposome, designed for the codelivery of *NBR1* small interfering RNA, a copper ionophore, and sorafenib restored FDX1-dependent cuproptosis and exhibited marked anti-HCC efficacy, suppressing HCC growth in vivo. **Conclusions:** MKIs suppressed SMYD2-mediated YTHDF1 methylation at K515 via the inactivation of AKT-mTOR signaling. This led to the inhibition of *FDX1* translation, resulting in AKT signaling reactivation and protein lipoylation impairment, effects that contributed to both MKI resistance and cuproptosis tolerance in HCC. Overcoming MKI resistance and resensitizing cells to cuproptosis by targeting NBR1-mediated YTHDF1 degradation using a nanoliposomal codelivery system represents a promising strategy for HCC treatment.

## Background

Hepatocellular carcinoma (HCC) is the most prevalent form of primary liver cancer, remaining a leading cause of cancer-related mortality worldwide due to its aggressive progression and limited therapeutic options [1]. While targeted therapies—primarily multikinase inhibitors (MKIs) such as sorafenib (Sora) and lenvatinib (Lenva)—have long been cornerstone treatments for advanced HCC, the therapeutic paradigm has recently shifted. Combination therapy with immune checkpoint inhibitors and angiogenesis inhibitors (including MKIs) has led to improved prognostic outcomes and is now recommended as the first-line intervention for this cancer [2]. However, the clinical benefits of these treatments are invariably curtailed by the rapid emergence of adaptive resistance [2–4]. Recent studies have implicated kinase rewiring, epigenetic plasticity, and tumor microenvironment remodeling as key drivers of this resistance [5–7]. Nevertheless, strategies aimed at simultaneously reversing resistance and reactivating tumor cell death pathways, particularly those dependent on metabolic vulnerabilities, remain underexplored. For example, cuproptosis, a recently identified copper-dependent form of regulated cell death triggered by mitochondrial protein lipoylation [8], represents a promising target in HCC, given that the liver is the major organ responsible for maintaining copper homeostasis in mammals [9–11]. Nevertheless, how cuproptosis is regulated under therapeutic stress, and its potential interplay with drug resistance in HCC, remain uncharacterized.

Mitochondrial ferredoxin 1 (FDX1) was recently identified as a critical determinant for cuproptosis induction [8,12]. It catalyzed the reduction of divalent copper ions (Cu^2+^) to the more toxic monovalent copper ions (Cu^+^) [8,12]. In addition, FDX1 directly interacted with lipoic acid synthetase, thereby enhancing its binding to glycine cleavage system H protein, a lipoyl carrier, ultimately increasing the lipoylation levels of proteins such as dihydrolipoamide S-acetyltransferase (DLAT) and dihydrolipoamide S-succinyltransferase (DLST). Copper overload induced the aggregation of lipoylated proteins, leading to irreversible mitochondrial dysfunction and cell death. Intriguingly, FDX1 also modulated kinase signaling networks important for cellular survival and growth [13], suggesting that it may play a dual role as a nexus influencing both cuproptosis sensitivity and MKI efficacy.

To this end, this study was designed to investigate the potential crosstalk between MKI resistance and cuproptosis tolerance in HCC. We aimed to identify the key node responsible for this concurrent adaptation and to delineate the regulatory network governing its function. Ultimately, our work sought to develop actionable strategies capable of simultaneously reversing adaptive resistance and enhancing cuproptosis induction.

## Materials and Methods

### Cell culture

The HCC cell lines MHCC-97H and Huh7, along with the human embryonic kidney cell line HEK293T, were purchased from the Cell Bank of the Typical Culture Preservation Committee, Chinese Academy of Sciences. The cell lines were authenticated via short tandem repeat genotyping. MHCC-97H, Huh7, and HEK293T cells were cultured in Dulbecco’s Modified Eagle’s medium with 100 U/ml penicillin and 100 μg/ml streptomycin (CR-12800-S, Cienry). All media were supplemented with 10% fetal bovine serum (FBS, 04-001-1ACS, Biological Industries).

Sora-resistant MHCC-97H cells (97H-SR) were generated by stepwise selection using 0.5 to 2.5 μmol/l Sora (S7397, Selleck) and were subsequently maintained at 2.5 μmol/l Sora for over 1 year. 97H-SR cells exhibited a marked increase in half-maximal inhibitory concentration for Sora compared with parental MHCC-97H cells (97H-P).

### Proteomic analysis

MHCC-97H cells were treated with 5 μmol/l Sora for 48 h. After cell pellet collection, quantitative proteomic analysis was conducted on the Astral DIA platform by Beijing Tsingke Biotech Co., Ltd.

### Cell treatment

To evaluate the dynamic changes in FDX1 protein and protein lipoylation levels including lipoylated DLAT and lipoylated DLST in response to MKI exposure, HCC cells were treated with 5 μmol/l Sora and 10 μmol/l Lenva (S1164, Selleck), and protein analysis was performed at different time points.

For cuproptosis sensitivity analysis, cells were treated with 5 to 100 nmol/l elesclomol (ES, HY-12040, MedChemExpress) or 50 to 500 nmol/l ditiocarb sodium (HY-B1637, MedChemExpress) diluted in culture medium supplemented with 1 μmol/l CuCl_2_ (C106774, Aladdin), and then submitted to cytotoxicity assays.

To examine changes in FDX1 protein stability after MKI treatment, cells were treated with 100 μg/ml cycloheximide (CHX, C7698, Sigma-Aldrich), and FDX1 protein levels were quantified at various time points.

To assess the effect of the pharmacological inhibition of YTH domain family protein 1 (YTHDF1) on FDX1 protein expression, cells were treated with 2 μmol/l tegaserod (Tega, HY-14153, MedChemExpress) for 24 h, followed by the quantification of FDX1 protein levels.

To assess the effect of methylation on YTHDF1 protein expression in the presence of Sora, cells were incubated with 10 μmol/l Sora with or without 1 μmol/l JIB-04 (S7281, Selleck) for 24 h, and YTHDF1 protein levels were subsequently quantified.

To investigate the mechanism underlying the MKI-regulated autophagic degradation of YTHDF1, cells were treated for 24 h with various receptor tyrosine kinase (RTK) inhibitors, comprising the vascular endothelial growth factor receptor (VEGFR) inhibitor tivozanib (0, 2, and 10 μmol/l; HY-10977, MedChemExpress), the Fms-related receptor tyrosine kinase 3 (FLT3) inhibitor FLT3-IN-3 (0, 2, and 10 μmol/l; HY-112145, MedChemExpress), the c-Kit inhibitor c-Kit-IN-1 (0, 5, and 10 μmol/l; HY-15240, MedChemExpress), and the platelet-derived growth factor receptor (PDGFR) inhibitor CP-673451 (0, 1, and 2 μmol/l, HY-12050, MedChemExpress). YTHDF1 protein levels were subsequently determined.

To examine the regulatory effect of the mitogen-activated protein kinase (MAPK) signaling pathway on YTHDF1, cells were treated with 100 μmol/l PD318088 (HY-12062, MedChemExpress) for 48 h, and changes in YTHDF1 expression were subsequently evaluated.

To assess the effect of protein kinase B (PKB, also known as AKT)-mechanistic target of rapamycin (mTOR) signaling pathway inhibition on YTHDF1, cells were treated with 10 μmol/l MK-2206 (HY-10358, MedChemExpress) or 20 μmol/l Rapamycin (HY-10219, MedChemExpress) for 48 h, after which YTHDF1 protein levels were quantified.

### Western blotting

Protein samples were thawed on ice, thoroughly vortexed, separated by sodium dodecyl sulfate-polyacrylamide gel electrophoresis, and transferred to membranes. After blocking with 5% (w/v) skimmed milk (FD0080, Fdbio Science) prepared in 1× Tris-buffered saline with 0.1% Tween 20 (TBS-T, FD0020, Fdbio Science) for 1 h at room temperature with gentle agitation, the membranes were incubated with the appropriately diluted primary antibodies (prepared according to the manufacturer’s instructions) overnight at 4 °C on a vertical rotator. The next day, the membranes were incubated with the corresponding species-specific secondary antibody (111-035-003, 115-035-003, Jackson) diluted in 5% skimmed milk for 1 h at room temperature. Signals were detected and imaged using an Amersham Imager 680 chemiluminescence imaging system (General Electric Company). The antibodies used in this study are detailed in Table S1. Each experiment was conducted with 3 independent biological replicates during the preliminary phase, and the results from one final formal experiment were selected for presentation.

### Mouse xenograft models

BALB/c nude mice (6 to 8 weeks old) were obtained from Shanghai Laboratory Animal Center and housed in a specific pathogen-free facility at the Laboratory Animal Research Center of Sir Run Run Shaw Hospital, Zhejiang University School of Medicine. The mice were housed under controlled conditions, comprising a 12-h/12-h light/dark cycle, a constant temperature of 22 ± 2 °C, and 50% ± 10% relative humidity, and had ad libitum access to standard rodent chow and water.

Several subcutaneous xenograft models were established in mice to evaluate the impact of treatments in vivo. To assess the effect of *FDX1* overexpression on Sora resistance, 1 × 10^7^ 97H-SR cells or 97H-SR cells overexpressing *FDX1* (97H-SR *FDX1*-oe) were subcutaneously injected into the right axilla of mice. Once the implanted tumors reached 50 to 300 mm^3^, tumor-bearing animals were randomly divided into 2 groups (*n* = 6 per group) and treated with dimethyl sulfoxide (DMSO, D2650, Sigma-Aldrich) or Sora (20 mg/kg) via intraperitoneal injection 4 times a week.

To evaluate the effect of Tega treatments on cuproptosis in vivo, 5 × 10^6^ MHCC-97H cells were subcutaneously injected into the right axilla of mice. When tumor volumes reached 50 to 300 mm^3^, mice were randomly allocated to 4 groups (*n* = 6 per group), in which the mice received intraperitoneal injections of DMSO, ES (50 mg/kg) + CuCl_2_ (0.06 mg/kg), Tega (1 mg/kg), or Tega (1 mg/kg) + ES (50 mg/kg) + CuCl_2_ (0.06 mg/kg) 4 times per week.

To investigate the in vivo effect of next to BRCA1 gene 1 protein (NBR1) on cuproptosis, 5 × 10^6^ MHCC-97H cells stably expressing either scrambled short hairpin RNA control (shNC) or short hairpin RNA targeting *NBR1* (sh*NBR1*) were subcutaneously injected into the right axilla of mice. When tumors reached 50 to 300 mm^3^, the mice were randomly assigned to 4 treatment groups (*n* = 6 per group) and administered the following regimens via intraperitoneal injection 4 times weekly: DMSO, Sora (10 mg/kg), ES (40 mg/kg) + CuCl_2_ (0.06 mg/kg), or Sora (10 mg/kg) + ES (40 mg/kg) + CuCl_2_ (0.06 mg/kg).

To determine the contribution of YTHDF1 lysine 515 (K515) methylation to the therapeutic response to combined targeted and cuproptosis-inducing therapy in vivo, subcutaneous tumors were established via the injection of 5 × 10^6^ MHCC-97H cells with knock-in of wild-type *YTHDF1* (*YTHDF1*-WT), *YTHDF1* with lysine 515 mutated to arginine (*YTHDF1*-K515R), or *YTHDF1* with lysine 515 mutated to methionine (*YTHDF1*-K515M) into the right axilla of mice. When the tumor volume reached 50 to 300 mm^3^, the mice were randomized into 2 groups (*n* = 6 each) and treated 4 times weekly with either DMSO or the combination of Sora (10 mg/kg) + ES (40 mg/kg) + 0.06 mg/kg CuCl_2_.

To evaluate the therapeutic efficacy of the super cuproptosis detonator liposome (SCDlip) in vivo, a subcutaneous xenograft model was established in BALB/c nude mice via the injection of 5 × 10^6^ Huh7 cells into the right axilla of the animals. Subsequently, the mice received tail vein injections 3 times per week of mock, siNClip that was coloaded with Sora, Cu^2+^-elesclomol (ES-Cu, HY-156376, MedChemExpress) and control small interfering RNA (siRNA), or SCDlip that was coloaded with Sora, ES-Cu, and *NBR1* siRNA.

Xenograft tumor volume was monitored using Vernier calipers. Tumor volume (mm^3^) was calculated using the formula 0.5 × tumor length × tumor width^2^. When the tumor volume in any mouse approached the maximum permitted size (1,500 mm^3^), the mouse was humanely euthanized by CO_2_ asphyxiation and the excised xenograft was imaged and weighed.

To further evaluate the therapeutic efficacy of the SCDlip in vivo, an orthotopic liver tumor model was established by injecting 5 × 10^5^ MHCC-97H luciferase-oe cells into the liver of BALB/c nude mice. After 1 week, the mice were allocated to receive one of the following treatments via tail vein injection, 4 times weekly: mock, siNClip, or SCDlip. D-luciferin potassium salt (40902ES02, YEASEN) was administered via intraperitoneal injection once weekly (150 mg/kg luciferin/body weight) and the luciferase signal intensity of the orthotopic liver tumors was recorded using a IVIS Lumina XRMS Series III small animal imaging system (Revvity). The experiment was terminated if mice exhibited sustained hypothermia, emaciation, dehydration, notable abdominal distension, or ascites. Finally, the xenografts obtained from the euthanized mice were imaged.

### Immunohistochemistry

For the xenograft tumor models in this study, all samples were fixed in 4% paraformaldehyde (G1101, Servicebio). Following fixation, the tissues were processed through a graded series of ethanol (10009218, SINOPHARM) and xylene (10023418, SINOPHARM), embedded in paraffin (DA CHUAN), and sliced into 3- to 5-μm sections. The samples were obtained from several nude mouse xenograft models: (a) MHCC-97H cell xenografts before and after Sora treatment (10 mg/kg) administered 5 times per week for 3 weeks (from our previous study [9]); (b) 97H-SR or 97H-SR *FDX1*-oe cell xenografts before and after Sora treatment; (c) xenografts derived from MHCC-97H cells stably expressing shNC or sh*NBR1* after treatment with DMSO, ES, Sora, or ES + Sora; (d) xenografts derived from MHCC-97H cells with the knock-in of *YTHDF1*-WT, *YTHDF1*-K515R, or *YTHDF1*-K515M before and after ES + Sora treatment; and (e) MHCC-97H cell orthotopic xenografts after treatment with SCDlip, siNClip, or control. The paraffin sections were deparaffinized using an eco-friendly deparaffinization agent (Tongsheng Technology), rehydrated through a graded series of anhydrous alcohol, and washed with Tris-buffered saline (TBS). Subsequently, antigen retrieval was performed in a pressure cooker using ethylenediaminetetraacetic acid (EDTA, 10009717, SINOPHARM) retrieval solution (pH 9), followed by cooling and washing in TBS. Then, the sections were soaked in 3% hydrogen peroxide (10011218, SINOPHARM) for 30 min. After blocking with 10% goat serum (AR1009, BOSTER) at room temperature for 30 min, the sections were incubated with indicated primary antibodies diluted in blocking solution at 4 °C overnight. After washing, the samples were incubated with the corresponding species-specific secondary antibodies (ab205718, Abcam) for 1 h at room temperature. Chromogenic detection was performed using 3,3′-diaminobenzidine staining (DAB4033, Maxim), and nuclei were counterstained with hematoxylin (BA4041, BASO). After dehydration with anhydrous alcohol, the sections were allowed to air-dry, mounted with an eco-friendly mounting medium (Tongsheng Technology), and imaged using a microscope (Olympus). Semi-quantitative analysis was performed using ImageJ (https://imagej.net/software/fiji/). The antibodies used are detailed in Table S1.

### Immunofluorescence staining for tissues

Paraffin-embedded tissue sections were deparaffinized in xylene and rehydrated through a graded ethanol series. Antigen retrieval was performed by heating the sections in EDTA buffer (pH 9) in a microwave or pressure cooker. After cooling and washing with TBS-T, the sections were blocked with 10% normal serum for 30 min at 37 °C, and then treated as follows: For DLAT detection alone, the sections were incubated with anti-DLAT primary antibody overnight at 4 °C. After washing with TBS-T, the sections were incubated with fluorophore-conjugated secondary antibody (ab150075, Abcam) for 45 min at 37 °C. For double staining of Ki-67 and FDX1, the sections were first incubated with anti-Ki-67 primary antibody overnight at 4 °C, followed by horseradish peroxidase (HRP)-conjugated secondary antibody (ab205718, Abcam) for 45 min at 37 °C, and then incubated with tyramide signal amplification (TSA)-488 tyramide (11060, AAT Bioquest) for 10 min at room temperature for TSA-based signal amplification. After washing and antibody stripping, the sections were incubated with anti-FDX1 primary antibody overnight at 4 °C, followed by HRP-conjugated secondary antibody (ab205718, Abcam) for 45 min at 37 °C, and then incubated with TSA-555 tyramide (11065, AAT Bioquest) for 10 min at room temperature for TSA-based signal amplification. For double staining of DLAT and Ki-67, after Ki-67 staining and antibody stripping using the same protocol as described for the Ki-67/FDX1 double staining, the sections were subjected to DLAT detection using anti-DLAT primary antibody overnight at 4 °C, followed by fluorophore-conjugated secondary antibody (ab150075, Abcam) for 45 min at 37 °C. Then, the sections were counterstained with 4′,6-diamidino-2-phenylindole (DAPI, C0060, Solarbio). Finally, the sections were mounted with an anti-fade mounting medium (0100-01, Southern Biotech) and imaged using a fluorescence microscope (Olympus). The antibodies used for immunofluorescence (IF) are detailed in Table S1.

### siRNA and plasmid transfections

Lipofectamine RNAiMAX transfection reagent (13778, Thermo Fisher Scientific) was used for siRNA transfections, while Lipo8000 Transfection Reagent (C0533, Beyotime) was employed for plasmid transfection. The siRNA duplexes were purchased from Genepharma and listed in Table S2.

The plasmid expressing *FDX1* (HG19339-NM) was purchased from SinoBiological and served as the template for the construction of pLVX-FDX1. Plasmids expressing SET and MYND domain-containing protein 2 (*SMYD2*) (P48617), Jumonji domain-containing protein 7 (*JMJD7*) (P5143), and *NBR1* (P53428) were purchased from Miaoling Biology. Flag-YTHDF1 and YTHDF1 truncations were gifts from Dr. Xinyang Hu from the Medical School of Zhejiang University. Flag-YTHDF1 was used as the template for the construction of Myc-YTHDF1. Full-length *NBR1* plasmid was used as the template for the construction of UBA-deletion mutant of *NBR1* (*NBR1*ΔUBA). The respective Myc-YTHDF1 mutants were constructed using the ClonExpress MultiS One Step Cloning Kit (C113) from Vazyme. Green fluorescent protein (GFP)-tagged LC3 was constructed based on the tandem monomeric red fluorescent protein (RFP)–GFP-tagged LC3 plasmid that we previously reported [14]. pCDH-luciferase plasmid was a gift from Dr. Yanan Xue from the Medical School of Zhejiang University.

For siRNA transfection, cells were grown in a multiwell plate to approximately 30% to 50% confluence. Before transfection, the medium was replaced with fresh antibiotic-free medium (CR-12800, Cienry) supplemented with 10% FBS. The transfection mixture was prepared using a 6-well plate format as a representative example. Two 1.5-ml EP tubes, labeled A and B, were prepared, each containing 250 μl of OPTI-MEM medium (CR-22600, Cienry). Tube A was supplemented with 2.5 to 7.5 μl of Lipofectamine RNAiMAX transfection reagent, while a specified amount of siRNA was added to Tube B, to a final concentration of 10 to 50 nmol/l. After mixing, both tubes were incubated at room temperature for 5 min. The contents of Tube B were then combined with those of Tube A, mixed gently, and further incubated for 20 min. The resulting mixture was carefully added dropwise into the culture wells. Following gentle shaking, the cells were incubated for 24 h after which the medium was replaced, and the cells were used for subsequent experiments. To investigate the role of mitochondrial proteases in the MKI-mediated modulation of FDX1, 2 key hydrolases previously identified [9] as being responsible for FDX1 protein turnover—AFG3 like matrix AAA peptidase subunit 2 (*AFG3L2*) and lon peptidase 1 (*LONP1*)—were simultaneously knocked down in HCC cells using siRNA to comprehensively inhibit the mitochondrial protease-dependent degradation of FDX1. The cells were then treated with 5 μmol/l Sora for 48 h, and then assessed for changes in FDX1 protein levels.

For plasmid transfection, cells were seeded in culture plates and allowed to grow to 30% to 50% confluence. Before transfection, the medium was replaced with fresh antibiotic-free medium containing 10% FBS. Using a 6-well plate as reference, the transfection complex was prepared by adding 2 μg of plasmid DNA and 4 μl of Lipo8000 Transfection Reagent to 200 μl of OPTI-MEM medium. The mixture was homogenized by gentle pipetting and was incubated at room temperature for 15 min. The complex was then carefully added to the culture medium. After gentle swirling, the cells were incubated for a further 24 h, after which the medium was replaced with fresh culture medium for subsequent experiments.

### Cell proliferation assay

The cell proliferation assay was performed using Cell Counting Kit-8 (CCK-8, 40203ES80, YEASEN). Briefly, following pretreatment (e.g., with drugs or siRNA knockdown), cells (4,000 per well) were seeded in a 96-well plate, allowed to adhere overnight, and then subjected to the indicated treatments. Subsequently, 200 μl of culture medium with 10% CCK-8 reagent was added to each well, and the plates were incubated for 30 to 60 min at 37 °C in a humidified atmosphere with 5% CO_2_. The absorbance of each well was measured at 450 nm using a microplate reader (Thermo Fisher Scientific). All samples were prepared in at least triplicate.

### Packaging of overexpression lentivirus and generation of stable cell lines

For the packaging of overexpression lentiviruses, the transfer plasmid pLVX-FDX1 or pCDH-luciferase was cotransfected with the packaging plasmids psPAX2 and pMD2.G into HEK293T cells. After 72 h, the culture supernatant was collected, the viruses were concentrated by ultracentrifugation at 120,000 × *g* for 2 h at 4 °C, aliquoted, and stored at −80 °C. To generate stable cell lines, the target cells (97H-SR *FDX1*-oe or MHCC-97H luciferase-oe) were infected with lentiviral particles for 48 h to allow for transgene expression. Subsequently, the infected cells were subjected to selective culture in complete medium supplemented with 2 μg/ml puromycin (J593-25MG, Amresco) for 7 days. The resulting puromycin-resistant populations, representing stable polyclonal cell lines, were used for subsequent functional experiments.

### IF assay for cell

Cells were seeded on coverslips and treated as indicated. These cells were then fixed in 4% paraformaldehyde for 10 min, permeabilized in 0.25% Triton X-100 (FD3511, Fdbio Science) diluted in 1× phosphate-buffered saline (PBS, CR-20012, Cienry) for 10 min, blocked with 3% bovine serum albumin (FD0030-500, Fdbio Science) diluted in 1× PBS for 1 h, and incubated with an anti-DLAT antibody (1:500) at 4 °C overnight. After washing 3 times with 0.1% PBS-T (PBS with 0.1% Tween 20), the cells were incubated with the appropriate secondary antibodies (a11029, Invitrogen) for 1 h at 37 °C, washed, and sealed with mounting medium containing DAPI (H-1200, Vectorlabs). Images were captured on a confocal microscope (Olympus). For mitochondrial staining, cells were incubated with 200 nmol/l Mitotracker Red CMXRos (M7512, Thermo Fisher Scientific) for 30 min, and then fixed in paraformaldehyde.

### Soluble–insoluble fraction isolation

Soluble–insoluble fraction isolation was performed as previously reported [9]. Following treatment, cells were lysed in ice-cold NP40 lysis buffer (pH 7.4) containing 20 mmol/l Tris (FD2010, Fdbio Science), 150 mmol/l NaCl (FD3467, Fdbio Science), 1% NP40 (FD7949, Fdbio Science), and an EDTA-free protease inhibitor cocktail (B14001, Selleck) for 30 min. The lysates were centrifuged at 21,000 × *g* for 30 min at 4 °C, and the supernatant and sediment fractions were separately collected. Each fraction was mixed with sodium dodecyl sulfate loading buffer (FD006, Fdbio Science), boiled, and sent for Western blotting.

### Detection of copper concentration

Copper ion detection was performed using the Cell Copper (Cu^2+^) Colorimetric Assay Kit from Elabscience (E-BC-K775-M). Briefly, cells were lysed using 200 μl of lysis buffer per 2 × 10^6^ cells, centrifuged at 12,000 × *g* for 30 min at 4 °C, and the supernatant was collected. A volume of 50 μl of the supernatant was used for the determination of protein concentration using a bicinchoninic acid assay (BCA) kit (P0010, Beyotime), and a volume of 100 μl of the supernatant was reacted with the 50-μl color development solution at 37 °C for 5 min, and absorbance was measured at 580 nm (*A*_580_). The copper content was calculated based on the *A*_580_ value and standard curve, and normalized to the protein concentration. The value was calculated according to the following formula:

Standard curve equation:y=ax+b (1)

$$\text{Copper}\;\mathrm{ion}\;\text{content}\;\left(\text{μmol}/\text{gprot}\right)=\left(\Delta A_{580}-b\right)÷a÷\mathrm{Cpr}$$ (2)

where *y* is the absorbance value of the standard well minus the absorbance value of the blank well (the absorbance value when the standard concentration is 0); *x* is the concentration of the standard; *a* is the slope of the standard curve; *b* is the intercept of the standard curve; Δ*A*_580_ is the measured absorbance value of the sample minus the absorbance value of the blank (the absorbance value when the standard concentration is 0); and Cpr is the protein concentration of the sample prior to being added to the detection system, in grams of protein per liter (gprot/liter).

### RNA extraction, reverse transcription, and quantitative polymerase chain reaction

Total RNA was isolated from cultured cells using TRIzon reagent (CW0580S, Cowin Bioscience). Briefly, cells were directly lysed in the culture plate for 5 min at room temperature. The resulting lysate was mixed with chloroform, and the mixture was centrifuged at 12,000 × *g* for 30 min at 4 °C to separate the phases. The aqueous phase was carefully transferred to a new tube, and RNA was precipitated by adding an equal volume of isopropanol. The RNA pellet was washed once with 75% ethanol, air-dried, and resuspended in RNase-free diethylpyrocarbonate water. RNA concentration and purity were determined using a NanoDrop 2000 spectrophotometer (Thermo Fisher Scientific). Reverse transcription (RT) was performed using the High-Capacity cDNA Reverse Transcription Kit (4368813, Applied Biosystems). For each reaction, 2 μg of total RNA was used as a template in a 20-μl reaction volume containing RT buffer, random primers, dNTP mix, and reverse transcriptase. The reaction was performed under the following conditions: 25 °C for 10 min, 37 °C for 2 h, and 85 °C for 5 min, and then held at 4 °C. The resulting cDNA was diluted and stored at −20 °C for subsequent quantitative polymerase chain reaction (qPCR) analysis. qPCR was undertaken in 20-μl reaction volumes containing 2 μl of diluted cDNA, 10 μl of 2× SYBR Green Master Mix (CW0957H, Cowin Bioscience), and 0.5 μmol/l of each forward and reverse primer. The amplification was conducted on a qPCR system with the following cycling conditions: an initial denaturation at 95 °C for 10 min, followed by 40 cycles of 95 °C for 10 s, 58 °C for 30 s, and 72 °C for 32 s. A standard melting curve analysis was then performed to confirm amplification specificity. Relative mRNA expression levels were calculated using the 2^−ΔΔCt^ method, with glyceraldehyde-3-phosphate dehydrogenase (*GAPDH*) serving as the internal control. Details of the primers used for qPCR are provided in Table S3.

### Polysome profiling

At approximately 80% confluence, treated MHCC-97H or Huh7 cells were mixed with 100 μg/ml CHX and incubated for 15 min. The cells were then lysed on ice for 15 min using poly-ribosome lysis buffer with the following composition: pH 7.4, 10 mmol/l 4-(2-hydroxyethyl)-1-piperazineethanesulfonic acid (E607018-0100, Sangon Biotech), 100 mmol/l KCl (P112144, Aladdin), 5 mmol/l MgCl_2_ (M283904, Aladdin), 100 μg/ml CHX, 1% Triton X-100, 1× RNase inhibitor (2313B, TaKaRa), 1 mmol/l dithiothreitol (FD0011, Fdbio Science), and 1× protease inhibitor, centrifuged at 20,000 × *g* for 10 min at 4 °C, and the supernatant was transferred to a 10% to 50% sucrose gradient and centrifuged at 288,000 × *g* for 130 min at 4 °C. Continuous monitoring was performed using the density gradient separation system (BioCamp) based on absorbance at 260 nm, and fractions were collected stepwise (approximately 0.5 ml per fraction). The RNA in each fraction was extracted using TRIzon reagent and the relative levels of *FDX1* mRNA in the ribosomes were analyzed by reverse transcription quantitative real-time polymerase chain reaction (RT-qPCR). Details of the primers used are given in Table S3.

### SunTag reporter system

The SunTag plasmid reporter system was constructed based on previous reports [15,16]. SunTag-related plasmids—pcDNA4TO-24×GCN4-v4-24×PP7 (P28968) and pHR-scFv-GCN4-sfGFP-GB1 (scFV-GFP, P26935)—were purchased from Miaoling Biology. The coding sequence and the 3′ untranslated region (3′-UTR) of *FDX1* were inserted into the pcDNA4TO-24×GCN4-v4-24×PP7 backbone to generate *FDX1*-SunTag plasmid. Cells were cotransfected with the *FDX1*-SunTag and scFV-GFP plasmids, and then treated as indicated. Finally, cells were seeded on coverslips, treated with 1 μg/ml doxycycline (HY-N0565, MedChemExpress) for 12 h, then sealed with mounting medium containing DAPI, and imaged using a confocal microscope (Olympus).

### *FDX1* N6-methyladenosine modification analysis

N6-methyladenosine (m6A) sequencing data for HCC cells were downloaded from the Gene Expression Omnibus database (https://www.ncbi.nlm.nih.gov/geo/) under accession GSE149510. The reads were aligned to the Human reference genome and (hg19) visualized using Integrative Genomics Viewer software (https://igv.org/).

### Screening of *FDX1* m6A regulatory proteins

To identify m6A-related proteins involved in the regulation of *FDX1*, a focused siRNA screen was performed in MHCC-97H and Huh7 cells. Key m6A regulators—the writers methyltransferase-like 3 (*METTL3*) and *METTL16*; the erasers fat mass and obesity-associated protein (*FTO*) and AlkB homolog 5 (*ALKBH5*); and the translation-related readers *YTHDF1*, YTH domain family protein 3 (*YTHDF3*), YTH domain containing 2 (*YTHDC2*), insulin-like growth factor 2 mRNA-binding protein 1 (*IMP1*), insulin-like growth factor 2 mRNA-binding protein 2 (*IMP2*), and insulin-like growth factor 2 mRNA-binding protein 3 (*IMP3*)—were individually knocked down using siRNA. At 48 h post-knockdown, protein was extracted and analyzed by Western blotting to assess the knockdown efficiency and evaluate changes in FDX1 protein levels.

### Methylated RNA immunoprecipitation

Following the siRNA-mediated knockdown of *METTL3* or *METTL16* in MHCC-97H or Huh7 cells, RNA was extracted using the TRIzon method. Methylated RNA immunoprecipitation (MeRIP) was performed according to the instructions of the EpiQuik CUT&RUN m6A RNA Enrichment Kit (P-9018-24, Epigentek). The extracted RNA was incubated with magnetic beads and an anti-m6A antibody (or control IgG) at room temperature for 90 min. After 3 washes followed by digestion with proteinase K at 55 °C for 15 min, the RNA was again extracted. The levels of m6A in *FDX1* mRNA were determined by RT-qPCR.

### RNA immunoprecipitation

RNA immunoprecipitation (RIP) was conducted using the PureBinding RIP Kit (P0102, Geneseed). Cells were lysed in lysis buffer, and the resulting lysate was incubated with magnetic beads conjugated with a primary anti-YTHDF1 antibody, with IgG serving as a control. Following incubation, the beads were washed 6 to 8 times. The RNA–protein complexes were subsequently eluted from the beads, and the bound RNA was purified and extracted. Finally, the binding of YTHDF1 to *FDX1* mRNA was quantified by RT-qPCR.

### Assessment of YTHDF1 degradation via the autophagy pathway

To determine the degradation pathway of YTHDF1 under MKI treatment, cells were treated first with 10 μmol/l Sora for 12 h, and then with 100 nmol/l bafilomycin A1 (Baf-A1, B1793, Sigma-Aldrich) or 10 μmol/l MG-132 (474790, Sigma-Aldrich) for 12 h, after which YTHDF1 protein levels were quantified. To further investigate whether YTHDF1 was degraded through the autophagic pathway, the key autophagy protein autophagy-related protein 5 (*ATG5*) and autophagy-related protein 7 (*ATG7*) were knocked down in HCC cells using siRNA. After 24 h of transfection, the cells were treated with Sora (5 μmol/l) for 48 h. The effect on YTHDF1 protein levels was then determined by Western blotting.

### Screening for autophagy adaptors for YTHDF1

To identify the autophagy adaptor responsible for YTHDF1 degradation under MKI treatment, a screening strategy focused on assessing the YTHDF1 protein recovery rate was implemented. A panel of candidate autophagy adaptor proteins [*NBR1*, sequestosome 1 (*SQSTM1*), optineurin (*OPTN*), Tax1 binding protein 1 (*TAX1BP1*), and nuclear dot protein 52 kDa (*NDP52*)] was first individually knocked down in HCC cells using siRNA for 24 h, after which the cells were treated with Sora (5 μmol/l) or Lenva (10 μmol/l) for 48 h. YTHDF1 protein levels were subsequently quantified by Western blotting.

### Immunoprecipitation/co-immunoprecipitation

For both immunoprecipitation (IP) and co-immunoprecipitation (co-IP), treated cells were lysed in Triton X-100 lysis buffer (50 mmol/l Tris-HCl, pH 7.4, 150 mmol/l NaCl, and 1% Triton-X-100) supplemented with a protease inhibitor cocktail. Following total protein quantification by BCA assay, 1 mg of the lysate was immunoprecipitated using the Flag-beads (A36797, Thermo Fisher Scientific), Myc-beads (HAK21044, Huabio), or agarose beads (78610, Thermo Fisher Scientific) conjugated to the indicated antibody. The resulting immunocomplex was washed, boiled, and analyzed by Western blotting.

### Short hairpin RNA construction and lentivirus packaging

DNA fragments corresponding to *NBR1* short hairpin RNA stem-loop structures were designed according to the cloning requirements of the pLKO.1-puro vector. The fragments were denatured at 95 °C and then allowed to cool naturally for annealing. Concurrently, the pLKO.1-puro vector was linearized via AgeI (R3552, NEW ENGLAND BioLabs) and EcoRI (R3101, NEW ENGLAND BioLabs) digestion, and then ligated to the annealed DNA fragments. The plasmid was then transformed into *E. coli* DH5α competent cells (9057, TaKaRa), and subsequently verified by sequencing to ensure correct plasmid construction (pLKO.1-sh*NBR1*). The psPAX2 and pMD2.G packaging plasmids and the constructed pLKO.1-sh*NBR1* plasmid were cotransfected into HEK293T cells. Subsequent steps for virus collection and concentration were performed as previously described for the pLVX-based overexpression system. The oligonucleotide sequences are listed in Table S4.

### Analysis of YTHDF1 posttranslational modifications under MKI treatment

HEK293T cells transiently transfected with a Flag-YTHDF1 plasmid were treated with 10 μmol/l Sora or an equivalent volume of DMSO for 24 h. Following treatment, the cells were lysed and subjected to IP using Flag-beads. To assess changes in lysine monomethylation (K-Me1), tyrosine phosphorylation (p-Tyr), serine/threonine phosphorylation (p-Ser/Thr), and lysine acetylation (K-Ace) on YTHDF1, IP eluates were then immunoblotted with antibodies specifically targeting these posttranslational modifications. To further investigate the role of acetylation in MKI-induced YTHDF1 down-regulation, MHCC-97H cells were treated with 1 mmol/l nicotinamide (NAM, S1899, Selleck) or 0.1 μmol/l trichostatin A (TSA, S1045, Selleck) for 24 h, and the protein levels of YTHDF1 were analyzed by Western blotting.

### Identification of critical K-Me1 modification sites in YTHDF1

To identify the critical lysine residue responsible for K-Me1 modification of YTHDF1, HEK293T cells were transfected with plasmids encoding Myc-tagged wild-type YTHDF1 or its methylation-inactive mutations (K371R, K419R, K515R, and K541R). At 48 h posttransfection, cells were collected and lysed, and the lysates were subjected to IP using anti-Myc beads. The K-Me1 levels were then determined by Western blotting.

### Domain mapping of the YTHDF1–NBR1 interaction

To map the domains involved in the interaction between YTHDF1 and NBR1, full-length YTHDF1 (FL) and its truncation variants (Δ1–46, Δ47–132, Δ133–252, Δ253–282, Δ283–539, and Δ540–560) were individually cotransfected with Myc-tagged, full-length NBR1 into HEK293T cells. At 48 h posttransfection, cells were harvested and lysed, and the lysates were subjected to co-IP using Myc-beads. The immunoprecipitated complexes and the total cell lysates (input) were then analyzed by Western blotting using anti-Flag (for YTHDF1) and anti-Myc (for NBR1) antibodies to determine which domains of YTHDF1 were necessary and sufficient for binding to NBR1.

### Molecular docking of YTHDF1 and NBR1

First, sequence information for the target proteins, NBR1 and YTHDF1, was retrieved from the UniProt database (https://www.uniprot.org/). The corresponding FASTA-format sequence files were downloaded and verified for integrity. The sequence files were then sequentially uploaded to the HDOCK server platform (http://hdock.phys.hust.edu.cn/) [17–21]. The “Protein–Protein Docking” mode was selected, and the docking calculation was initiated using the server’s default parameter settings. The server automatically predicted the 3-dimensional structures of the target proteins and simulated the binding conformations. Upon completion, files containing the docking results were downloaded in Protein Data Bank format. Finally, PyMOL software (https://www.pymol.org/pymol.html) was employed for structural analysis and visualization.

### Generation of a YTHDF1 K515 monomethylation antibody

A polyclonal antibody specific for YTHDF1 K515 monomethylation (K515-Me1) was generated by immunizing New Zealand White rabbits (Hangzhou Yuhang Kelian Rabbit Farming Professional Cooperative) with the Keyhole Limpet Hemocyanin-conjugated synthetic peptide CQEVPLEK(me1)AKQVL-C (Huabio). After multiple immunizations, serum titers were regularly monitored by indirect enzyme-linked immunosorbent assay. Once the titer reached the predetermined threshold, final bleeds were performed. The collected antiserum was subsequently purified using an affinity chromatography column containing the immobilized methylated peptide to obtain a highly specific polyclonal antibody. The specificity of the antibody was validated by Western blotting, which demonstrated its ability to recognize YTHDF1 WT while showing no cross-reactivity with YTHDF1-K515R or YTHDF1-K515M mutants.

### CRISPR-Cas9-mediated knock-in cell construction

Two guide RNAs (gRNAs) targeting *YTHDF1* (NCBI ID: 54915) were designed using the GenePharma online design software and were constructed within the pGE-4 (pU6gRNA1U6gRNA2Cas9puro) plasmid system (GenePharma). Additionally, 2 donor sequences were designed to provide repair templates for generating the K515R and K515M mutations in *YTHDF1*, and were individually cloned into the pUC57 vector. Subsequently, the gRNAs and donor sequences were cotransfected into pre-plated liver cancer cells at a ratio of 3:1. After 24 h, cells were selected using 2 μg/ml puromycin. Surviving cells were diluted, and single cells were seeded in a 96-well plate. Once a clone reached 100% confluence, 5,000 cells were subjected to polymerase chain reaction (PCR) followed by Sanger sequencing to verify that the desired mutations had been successfully introduced. The sequences of the gRNAs and donors are listed in Table S5.

### Assessment of the effect of SMYD2 inhibition on YTHDF1 methylation

To determine the effect of SMYD2 pharmacological inhibition on YTHDF1 K515-Me1, HEK293T cells overexpressing Myc-YTHDF1 were treated with 5 μmol/l LLY-507 (S7575, Selleck) for 48 h. Subsequently, IP was performed using Myc-beads, and the K515-Me1 levels in Myc-YTHDF1 were determined by Western blotting.

### Synthesis and in vivo distribution of SCDlip

For the development of SCDlip, the synthesized *NBR1* siRNA was labeled with the Cy5 fluorescent dye for in vivo tracing and featured a 2′-O-methoxyethyl (2′-OME) modification to enhance its stability and therapeutic efficacy by Genepharma. Both the administered siNClip and SCDlip formulations contained a base drug mixture consisting of 5 mg/kg Sora and 5 mg/kg ES-Cu, with siNClip incorporating 1.5 mg/kg siNC siRNA and SCDlip incorporating 1.5 mg/kg *NBR1* #1 siRNA. The liposome carrier was composed of [(4-hydroxybutyl)azanediyl]bis(hexane-6,1-diyl)bis(2-hexyldecanoate) (81000226, Tanshtech), 1,2-distearoyl-sn-glycero-3-phosphocholine (81000047, Tanshtech), cholesterol (81000048, Tanshtech), and 2-[(polyethylene glycol)-2000]-N,N-ditetradecylacetamide (81000227, Tanshtech) and was synthesized by GuangZhou Tanshtech Co., Ltd.

A total of 5 × 10^6^ MHCC-97H cells were used to establish subcutaneous xenograft tumors in the right axilla of nude mice. After 3 weeks, normal saline or Cy5-labeled SCDlip was administered to the mice via tail vein injection. After 5 min, the distribution of SCDlip was detected using a IVIS Lumina XRMS Series III small animal imaging system (Revvity). Subsequently, the mice were humanely euthanized via CO_2_ asphyxiation, and the heart, liver, spleen, lungs, and subcutaneous tumor were isolated. The distribution of SCDlip in these organs was then examined again using the same imaging system.

### Statistical analysis

The Student *t* test was used for comparisons between 2 groups. One-way analysis of variance and 2-way analysis of variance were employed for comparisons among 3 or more groups. The data were presented as means ± SD. A *P* value of less than 0.05 was considered statistically significant for all experiments.

## Results

### FDX1 down-regulation conferred resistance to MKIs in HCC

**Fig. 1.**
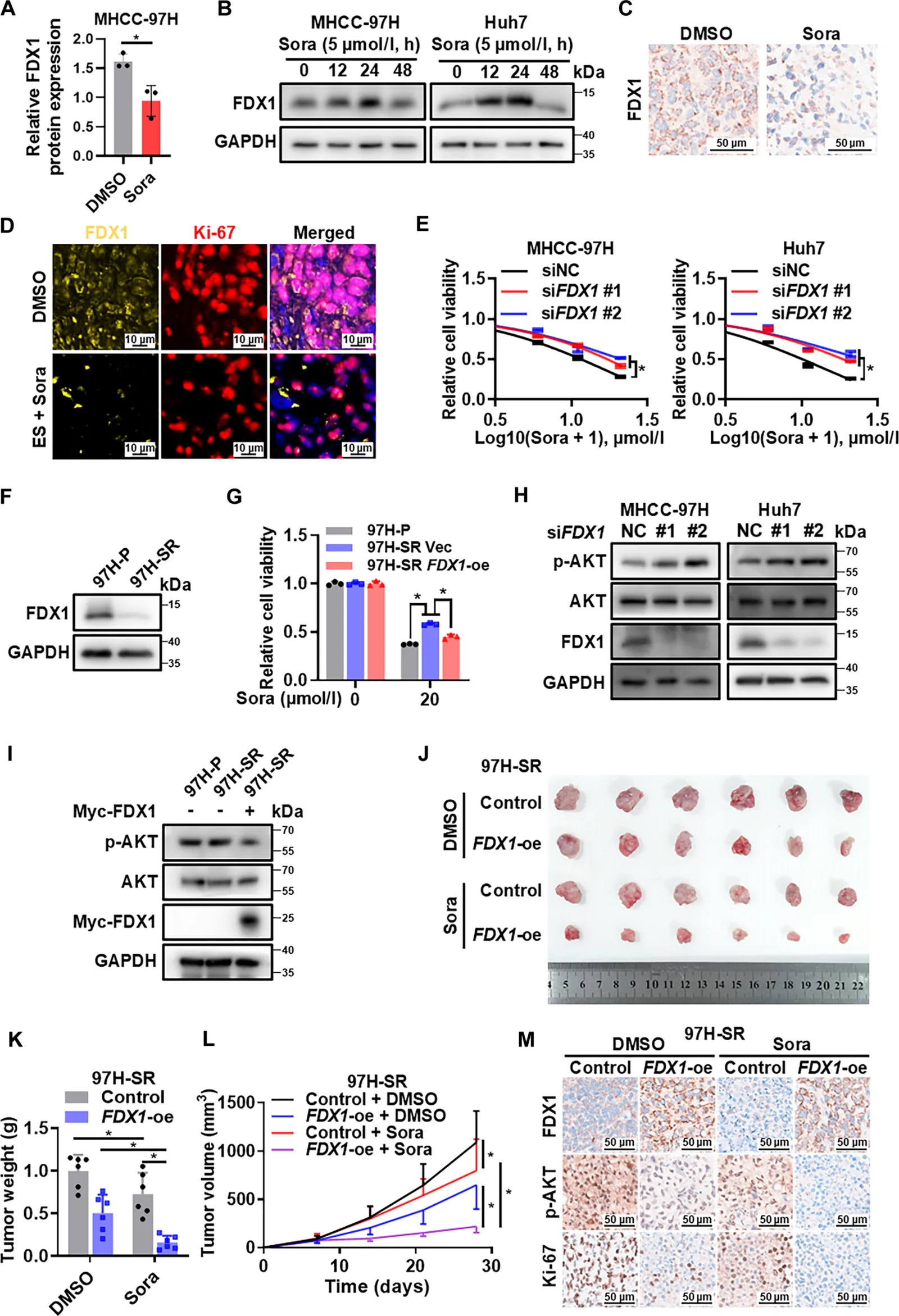
FDX1 down-regulation conferred resistance to MKIs in HCC. (A) After 5 μmol/l Sora treatment for 48 h in MHCC-97H cells, FDX1 protein levels were assessed by quantitative proteomic analysis. (B) The time course effect of 5 μmol/l Sora on FDX1 was analyzed by Western blotting in MHCC-97H and Huh7 cells. (C) Each group of xenograft tumor sections comprising DMSO and Sora were stained with FDX1 by IHC. (D) Tissue sections from the DMSO and ES + Sora group were labeled with FDX1 (yellow), Ki-67 (red), and DAPI (blue). (E) After *FDX1* knockdown, cell viability of MHCC-97H and Huh7 cells under Sora treatment for 48 h was measured with CCK-8 assay. (F) The protein levels of FDX1 in 97H-P and 97H-SR were determined by Western blotting. (G) Sora was added for 48 h in 97H-P, 97H-SR Vec or 97H-SR *FDX1*-oe. Cell viability was measured with CCK-8 assay. (H) Western blotting analysis of p-AKT, AKT, and FDX1 after *FDX1* knockdown in MHCC-97H and Huh7 cells. (I) Western blotting analysis of p-AKT, AKT, and Myc-FDX1 in 97H-P, 97H-SR Vec, or 97H-SR *FDX1*-oe. (J to L) Nude mice xenograft model (*n* = 6 per group) was generated by subcutaneous inoculation of 97H-SR or 97H-SR *FDX1*-oe. Mice were then treated with indicated drugs comprising DMSO and Sora 4 times a week. Tumor pictures (J), weight (K), and growth curve (L) were summarized and shown respectively. (M) Each group of xenograft tumor sections was stained with FDX1, p-AKT, and Ki-67 by IHC. Abbreviations: MKIs, multikinase inhibitors; HCC, hepatocellular carcinoma; Sora, sorafenib; IHC, immunohistochemistry; ES, elesclomol; CCK-8, Cell Counting Kit-8; 97H-P, parental MHCC-97H cells; 97H-SR, sorafenib resistant MHCC-97H cells; Vec, vector; *FDX1*-oe, overexpression of *FDX1*.

We previously found that transient Sora treatment (less than 24 h) could block the mitochondrial degradation of FDX1 [9]. However, quantitative proteomic analysis revealed that FDX1 was markedly down-regulated in HCC cells subjected to long-term Sora treatment (48 h) (Fig. 1A). To clarify the effect of Sora on FDX1, we first determined FDX1 expression in HCC cells following prolonged exposure to Sora and Lenva, both MKIs. Notably, while FDX1 protein levels showed a transient increase with short-term Sora treatment, they were down-regulated after 48 h (Fig. 1B). Lenva also led to down-regulation of FDX1 without the transient increase (Fig. S1A). Additionally, the expression of FDX1 was greatly reduced in HCC xenograft tissues with Sora treatment (Fig. 1C). Notably, FDX1 expression was negatively associated with active HCC cell proliferation after ES and Sora cotreatment. Tumor cells expressing FDX1 displayed weak Ki-67 staining, whereas the absence of the FDX1 signal was related with high Ki-67 expression (Fig. 1D). Furthermore, HCC cells with *FDX1* knockdown exhibited considerable tolerance to Sora and Lenva (Fig. 1E and Fig. S1B). Consequently, we next measured FDX1 protein expression in 97H-P and 97H-SR cells and found that it was indeed reduced in 97H-SR cells (Fig. 1F). In contrast to the vector control, the overexpression of *FDX1* restored Sora sensitivity in resistant HCC cells (Fig. 1G). The down-regulation of FDX1 has been reported to promote the activation of the AKT signaling pathway, enabling cancer cell proliferation [13]. Here, we observed that *FDX1* knockdown enhanced AKT activation, while its overexpression suppressed AKT signaling in Sora-resistant cells (Fig. 1H and I). In vivo, *FDX1* overexpression restored Sora efficacy in Sora-resistant HCC cells (Fig. 1J to M). Taken together, these data indicated that FDX1 down-regulation rendered HCC cells resistant to MKIs through the reactivation of AKT survival signaling.

### MKIs compromised cuproptosis induction by down-regulating FDX1 expression

**Fig. 2.**
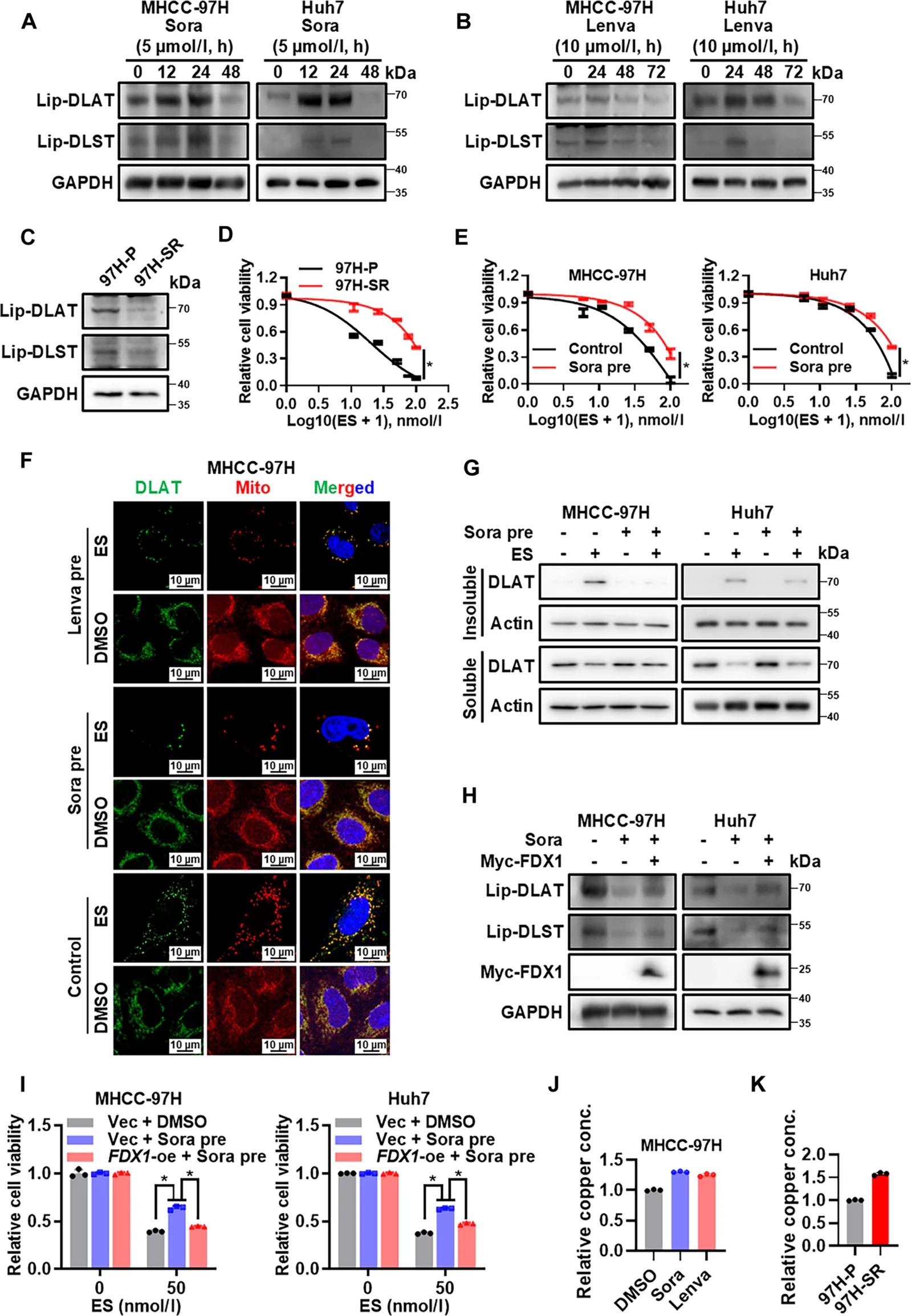
MKIs compromised cuproptosis induction by down-regulating FDX1 expression. (A and B) The levels of Lip-DLAT and Lip-DLST in MHCC-97H and Huh7 cells under time course effect of 5 μmol/l Sora or 10 μmol/l Lenva treatment were analyzed by Western blotting. (C) The levels of Lip-DLAT and Lip-DLST in 97H-P and 97H-SR were determined by Western blotting. (D) Cell viability of 97H-P and 97H-SR with 48-h ES treatment was measured by CCK-8. (E) After 48-h 5 μmol/l Sora pretreatment, controlled with DMSO, cell viability of MHCC-97H and Huh7 cells under ES and Sora treatment for 48 h was measured with CCK-8 assay. (F) After 5 μmol/l Sora or 10 μmol/l Lenva pretreatment for 48 h in MHCC-97H cells, 25 nmol/l ES was added for 48 h. DLAT aggregation was analyzed by IF (green, DLAT; red, Mitotracker; blue, DAPI). (G) After 5 μmol/l Sora pretreatment for 48 h in MHCC-97H and Huh7 cells, 25 nmol/l ES was added for 48 h. DLAT aggregation was analyzed by soluble–insoluble fraction isolation. (H) Western blotting analysis of the expression of Lip-DLAT and Lip-DLST with or without *FDX1* overexpression after 5 μmol/l Sora treatment for 48 h in MHCC-97H and Huh7 cells. (I) MHCC-97H and Huh7 cells were transfected with Myc-FDX1, controlled with empty vector, then pretreated with 5 μmol/l Sora for 48 h and DMSO was used as a control. After that, ES (50 nmol/l, controlled with DMSO) was further added 48 h, and cell viability was measured by CCK-8 assay. (J) The concentration of copper after 5 μmol/l Sora or 10 μmol/l Lenva with 10 nmol/l ES treatment for 48 h in MHCC-97H cells was measured by ELISA. (K) The concentration of copper within 97H-P and 97H-SR under 10 nmol/l ES incubation for 24 h was measured by ELISA. Abbreviations: MKIs, multikinase inhibitors; Lip-DLAT, lipoylated DLAT; Lip-DLST, lipoylated DLST; Sora, sorafenib; 97H-P, parental MHCC-97H cells; 97H-SR, sorafenib resistant MHCC-97H cells; ES, elesclomol; pre, pretreatment; CCK-8, Cell Counting Kit-8; IF, immunofluorescence; Vec, vector; *FDX1*-oe, overexpression of *FDX1*; Lenva, lenvatinib; ELISA, enzyme-linked immunosorbent assay; Conc, concentration.

Given that FDX1 played a critical role in promoting protein lipoylation and subsequent cuproptosis [8], we speculated that prolonged exposure to MKIs may suppress FDX1 expression, thereby compromising cuproptosis induction. Indeed, we noted that protein lipoylation including lipoylated DLAT and lipoylated DLST was decreased in both HCC cells subjected to long-term MKI treatment and Sora-resistant HCC cells (Fig. 2A to C). In addition, high concentrations of MKI also reduced the levels of lipoylated DLAT and lipoylated DLST (Fig. S2A and B). Moreover, Sora-resistant HCC cells were more resistant to cuproptosis induction by the copper ionophore ES than parental cells (Fig. 2D). Similarly, pretreatment with MKIs compromised cuproptosis sensitivity and reduced DLAT aggregation—a foremost hallmark of cuproptosis, which is formed by Cu^+^ binding to lipoylated DLAT during cuproptosis induction [8] (Fig. 2E to G and Fig. S2C to E). Importantly, in contrast to the vector control, exogenous *FDX1* application effectively reversed the MKI-induced inhibition of protein lipoylation and cuproptosis tolerance (Fig. 2H and I and Fig. S2F and G). Furthermore, intracellular copper concentrations were not reduced after MKI treatment or in Sora-resistant HCC cells, but were instead slightly elevated (Fig. 2J and K). These results confirmed that MKIs down-regulated FDX1 expression, thereby compromising cuproptosis induction in HCC cells.

### MKIs retarded *FDX1* translation in a YTHDF1-dependent manner

**Fig. 3.**
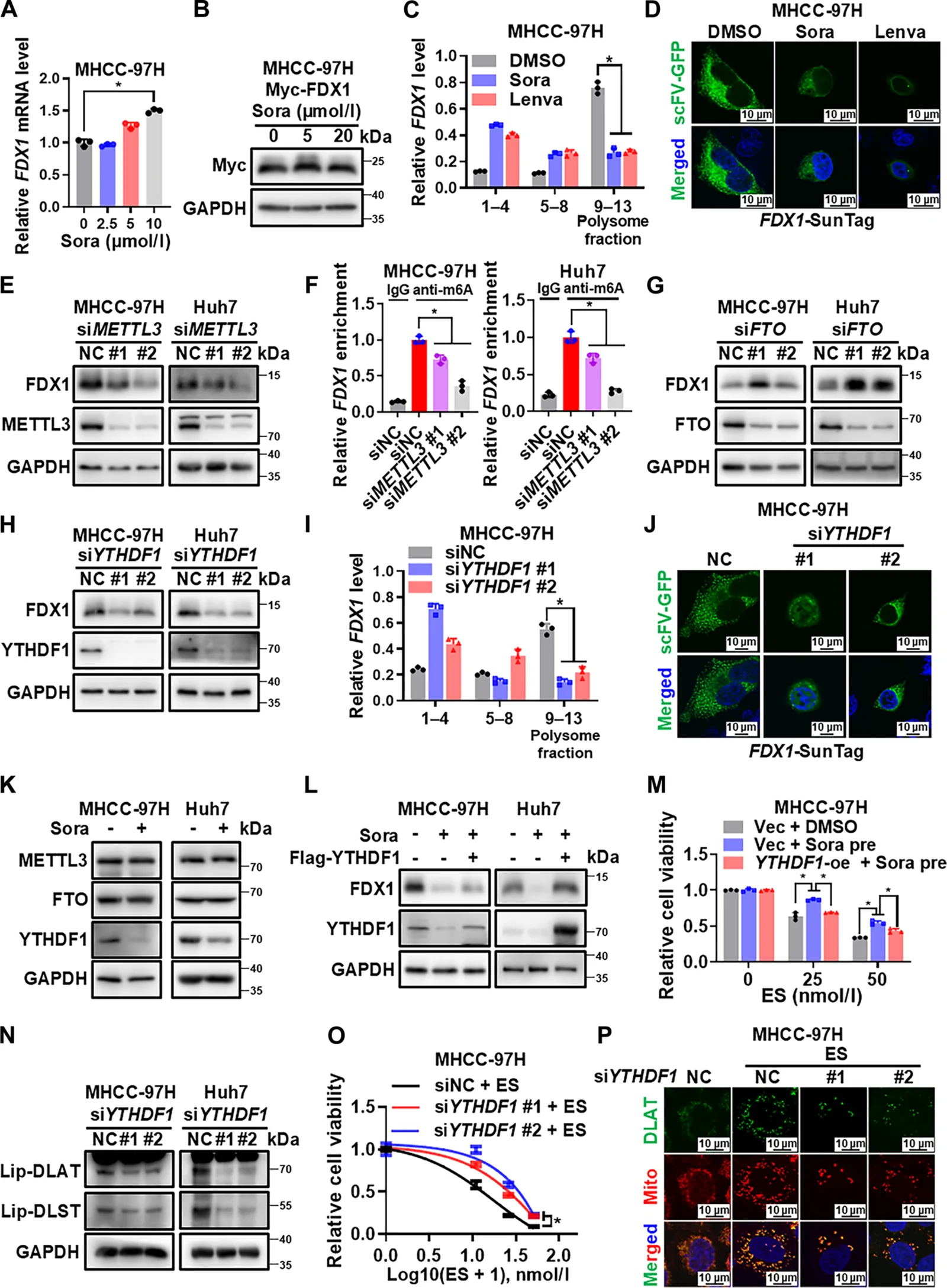
MKIs delayed FDX1 translation in a YTHDF1-dependent manner. (A) *FDX1* mRNA levels under Sora 48 h treatment in MHCC-97H cells were analyzed by RT-qPCR. (B) Myc-FDX1 overexpressed MHCC-97H cells were incubated with Sora for 48 h, and Western blotting was performed to detect the protein levels of Myc-FDX1. (C) Relative amount of *FDX1* mRNA in various polysome fractions under 5 μmol/l Sora or 10 μmol/l Lenva for 48 h was analyzed by RT-qPCR in MHCC-97H cells. Fractions 1 to 4 corresponded to the 40S and 60S ribosomal subunits, fractions 5 to 8 represented the monosome fraction, and fractions 9 to 13 indicated the polysome fraction. (D) MHCC-97H cells were cotransfected with *FDX1*-SunTag and scFv-GFP reporter plasmids, then 5 μmol/l Sora or 10 μmol/l Lenva was added for 48 h, controlled with DMSO. Newly synthesized FDX1 peptide signals were detected by IF. (E) Western blotting analysis of FDX1 after *METTL3* knockdown in MHCC-97H or Huh7 cells. (F) m6A levels of *FDX1* mRNA after *METTL3* knockdown in MHCC-97H and Huh7 cells were detected by MeRIP. (G) Western blotting analysis of FDX1 after *FTO* knockdown in MHCC-97H or Huh7 cells. (H) Western blotting analysis of FDX1 after *YTHDF1* knockdown in MHCC-97H or Huh7 cells. (I) Relative amount of *FDX1* mRNA in various gradient fractions after *YTHDF1* knockdown was analyzed by RT-qPCR in MHCC-97H cells. Fractions 1 to 4 corresponded to the 40S and 60S ribosomal subunits, fractions 5 to 8 represented the monosome fraction, and fractions 9 to 13 indicated the polysome fraction. (J) MHCC-97H cells were cotransfected with *FDX1*-SunTag, scFv-GFP reporter plasmid, and siNC/si*YTHDF1*. Newly synthesized FDX1 peptide signals were detected by IF. (K) METTL3, FTO, and YTHDF1 protein levels after 5 μmol/l Sora treatment for 48 h were analyzed by Western blotting in MHCC-97H or Huh7 cells. (L) The effect of Flag-YTHDF1 overexpression on FDX1 protein levels under 5 μmol/l Sora treatment for 48 h was analyzed by Western blotting in MHCC-97H or Huh7 cells. (M) MHCC-97H cells were transfected with Flag-YTHDF1/vector, then pretreated with 5 μmol/l Sora for 48 h. After that, ES was added for another 48 h, and cell viability was measured by CCK-8 assay. (N) The levels of Lip-DLAT and Lip-DLST after *YTHDF1* knockdown were analyzed by Western blotting in MHCC-97H or Huh7 cells. (O) After *YTHDF1* knockdown in MHCC-97H cells, ES was added for 48 h, and cell viability was measured with CCK-8 assay. (P) MHCC-97H cells were transfected with si*YTHDF1* or siNC, and 25 nmol/l ES was added for 48 h. DLAT aggregation was analyzed by IF (green, DLAT; red, Mitotracker; blue, DAPI). Abbreviations: MKIs, multikinase inhibitors; Sora, sorafenib; RT-qPCR, reverse transcription quantitative real-time polymerase chain reaction; Lenva, lenvatinib; IF, immunofluorescence; MeRIP, methylated RNA immunoprecipitation; Vec, vector; *YTHDF1*-oe, overexpression of YTHDF1; ES, elesclomol; CCK-8, Cell Counting Kit-8.

Subsequently, we analyzed the mechanism underlying the MKI-induced down-regulation of FDX1. Knocking down *AFG3L2* and *LONP1*, previously identified mitochondrial proteases responsible for FDX1 degradation [9], failed to prevent the down-regulation of FDX1 induced by Sora treatment (Fig. S3A). Additionally, neither Sora nor Lenva treatment decreased the levels of *FDX1* mRNA (Fig. 3A and Fig. S3B). MKI treatment also did not decrease exogenous FDX1 expression (Fig. 3B and Fig. S3C), nor did it impair FDX1 protein stability, instead enhancing it (Fig. S3D). In contrast, polysome profiling showed that MKI treatment caused a redistribution of *FDX1* mRNA from the heavy polysome fractions to lighter monosome (80S) and subunit (40S/60S) fractions (Fig. 3C and Fig. S3E), which was indicative of stalled translation initiation and reduced translation efficiency. Using the SunTag translation reporter system, we also observed that MKI administration markedly reduced the number of GFP puncta (Fig. 3D and Fig. S3F), indicating that MKIs suppressed *FDX1* mRNA translation in HCC cells.

As m6A modification plays a crucial role in the regulation of mRNA translation [22], we wondered whether MKI treatment would inhibit the translation of *FDX1* mRNA in an m6A modification-dependent manner. Analysis of the public dataset GSE149510 showed that *FDX1* mRNA has a high abundance of m6A modifications in its 3′-UTR (Fig. S3G). A focused screen of major m6A methyltransferases, demethylases, and reader proteins [22,23] indicated that si*METTL3*, but not si*METTL16*, down-regulated FDX1 protein expression (Fig. 3E and Fig. S3H), and decreased its mRNA m6A levels (Fig. 3F and Fig. S3I). Conversely, si*FTO*, but not si*ALKBH5*, increased FDX1 protein abundance (Fig. 3G and Fig. S3J). Among the m6A reader proteins assessed, we found that only si*YTHDF1* down-regulated FDX1 protein levels (Fig. 3H and Fig. S3K to O). Additionally, treatment with the YTHDF1 inhibitor Tega [24] effectively suppressed the protein expression of FDX1 (Fig. S3P). Meanwhile, there was a decline in the interaction between YTHDF1 and *FDX1* mRNA when *METTL3* was knocked down (Fig. S3Q). Furthermore, both polysome profiling experiments and SunTag reporter assays confirmed that the knockdown of either *YTHDF1* or *METTL3* effectively inhibited *FDX1* translation (Fig. 3I and J and Fig. S4A to D). These findings confirmed that *FDX1* translation was regulated by mRNA m6A modification, with METTL3, FTO, and YTHDF1 serving as writer, eraser, and reader, respectively.

Next, we explored how *FDX1* mRNA translation was affected by MKI treatment. Exposure to MKIs did not down-regulate *FDX1* mRNA m6A levels (Fig. S4E). Consistent with this, the protein levels of METTL3 and FTO remained unchanged, whereas YTHDF1 protein levels were markedly reduced after MKI treatment (Fig. 3K and Fig. S4F), while exogenous YTHDF1 replenishment effectively reversed MKI-induced FDX1 down-regulation and subsequent tolerance to cuproptosis (Fig. 3L and M and Fig. S4G and H).

Knocking down *YTHDF1* decreased protein lipoylation (Fig. 3N) and impaired cuproptosis sensitivity (Fig. 3O and Fig. S5A and B), effects that were reversed through the administration of exogenous *FDX1* (Fig. S5C). In addition, the YTHDF1 inhibitor Tega also reduced the susceptibility of liver cancer cells to cuproptosis (Fig. S5D). Both *YTHDF1* knockdown and Tega treatment effectively disrupted ES-induced DLAT aggregation (Fig. 3P and Fig. S5E to H), while Tega administration greatly curtailed the antitumor efficacy of ES in vivo (Fig. S5I to L). Collectively, these results established YTHDF1 as an essential mediator of cuproptosis. In summary, our data indicated that MKIs inhibited *FDX1* mRNA translation by reducing the abundance of YTHDF1, thereby contributing to cuproptosis tolerance in HCC.

### MKIs triggered the NBR1-mediated autophagic degradation of YTHDF1

**Fig. 4.**
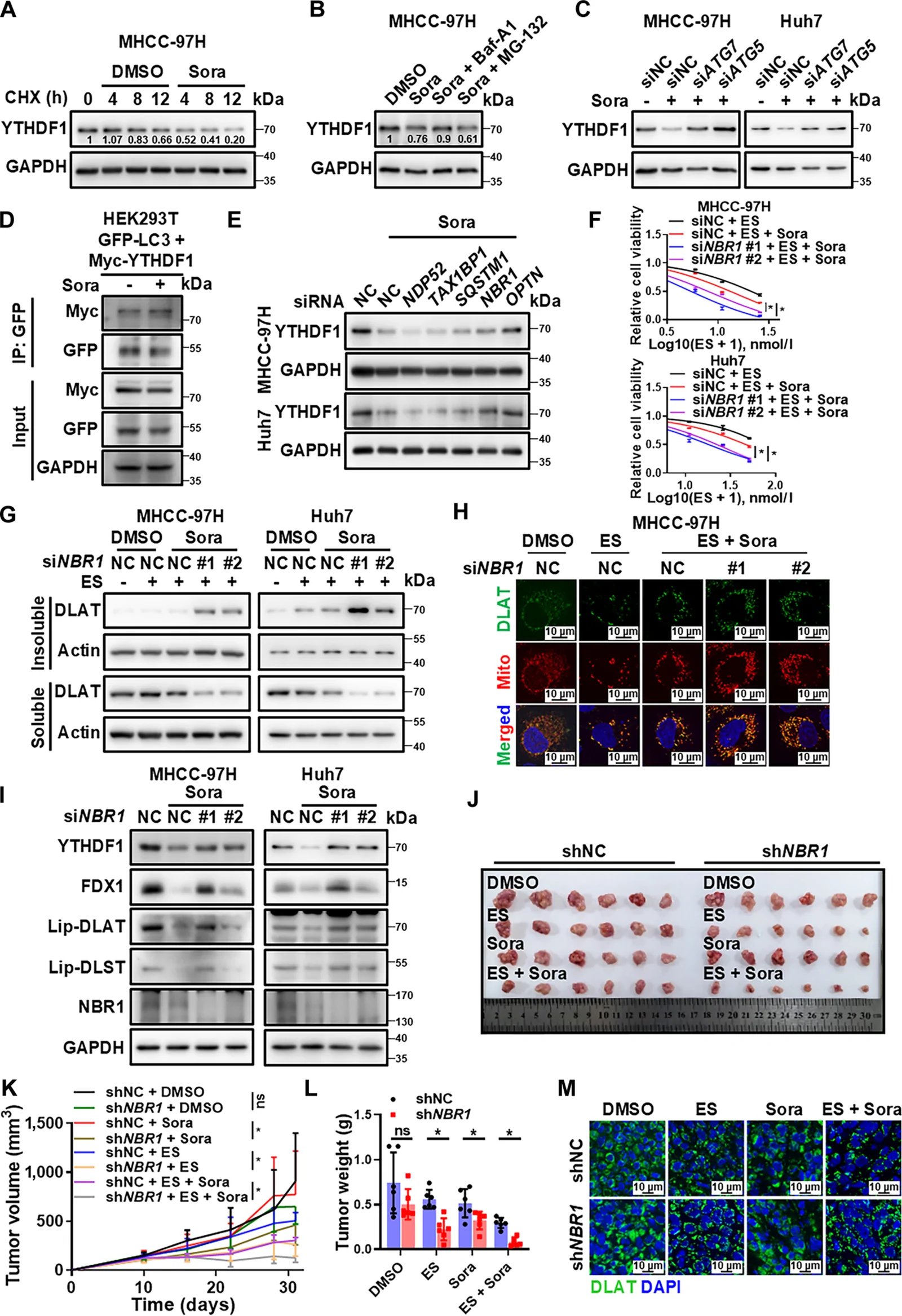
MKIs triggered the NBR1-mediated autophagic degradation of YTHDF1. (A) MHCC-97H cells were incubated with 10 μmol/l Sora and 100 μg/ml CHX for the indicated time and YTHDF1 protein stability was analyzed by Western blotting. (B) MHCC-97H cells were first incubated with 10 μmol/l Sora for 12 h, together with 100 nmol/l Baf-A1 or 10 μmol/l MG-132 for another 12 h, controlled with DMSO. The YTHDF1 protein levels were analyzed by Western blotting. (C) MHCC-97H or Huh7 cells were transfected with siNC, si*ATG5*, or si*ATG7*, then treated with 5 μmol/l Sora for 48 h. The YTHDF1 protein levels were analyzed by Western blotting. (D) The interaction between GFP-LC3 and Myc-YTHDF1 in HEK293T cells with or without 10 μmol/l Sora treatment for 24 h was analyzed by co-IP. (E) After MHCC-97H or Huh7 cells were transfected with siRNA of indicated autophagy adaptors respectively, 5 μmol/l Sora was added for 48 h, controlled with DMSO. The protein levels of YTHDF1 were analyzed by Western blotting. (F) After *NBR1* knockdown in MHCC-97H and Huh7 cells, 5 μmol/l Sora was added for 48 h, together with ES, and cell viability was measured with CCK-8 assay. (G) After *NBR1* knockdown in MHCC-97H or Huh7 cells, cells were treated with 25 nmol/l ES for 48 h, together with 5 μmol/l Sora, controlled with DMSO. DLAT aggregation was analyzed by soluble–insoluble fraction isolation. (H) After *NBR1* knockdown in MHCC-97H cells, cells were treated with 25 nmol/l ES for 48 h, together with 5 μmol/l Sora, controlled with DMSO. DLAT aggregation was analyzed by IF (green, DLAT; red, Mitotracker; blue, DAPI). (I) The *NBR1* knockdown MHCC-97H and Huh7 cells were treated with 5 μmol/l Sora for 48 h, controlled with DMSO. The protein levels of YTHDF1, FDX1, Lip-DLAT, and Lip-DLST were analyzed by Western blotting. (J to L) Nude mice xenograft model (*n* = 6 per group) was generated by subcutaneous inoculation of shNC/sh*NBR1* cells. Mice were then treated with indicated drugs 4 times a week comprising DMSO, ES, Sora, and Sora + ES. Tumor pictures (J), growth curve (K) and weight (L) were summarized and shown respectively. (M) Tissue sections from each group of shNC/sh*NBR1* xenograft tumors were labeled with DLAT (green) and DAPI (blue) by IF. Abbreviations: MKIs, multikinase inhibitors; Sora, sorafenib; CHX, cycloheximide; Baf-A1, bafilomycin A1; co-IP, co-immunoprecipitation; ES, elesclomol; IF, immunofluorescence; Lip-DLAT, lipoylated DLAT; Lip-DLST, lipoylated DLST; ns, not statistically significant.

We further explored the mechanisms by which MKIs induced YTHDF1 down-regulation. We found that MKI treatment led to an increase, rather than a decline, in *YTHDF1* mRNA levels (Fig. S6A), indicating that the MKI-induced reduction in YTHDF1 protein levels did not occur via mRNA down-regulation. In contrast, MKI treatment markedly decreased the protein levels of exogenously administered YTHDF1 (Fig. S6B). Additionally, MKI exposure was observed to accelerate YTHDF1 protein turnover after CHX treatment (Fig. 4A), suggesting that MKIs promoted YTHDF1 degradation. Interestingly, Baf-A1, an inhibitor of lysosomal protein degradation, reversed the MKI-induced decrease in YTHDF1 protein levels, whereas the MG-132-mediated inhibition of the ubiquitin–proteasome pathway did not (Fig. 4B). Moreover, knocking down *ATG5* or *ATG7*, 2 key autophagy-related proteins, also reversed the MKI-induced decline in YTHDF1 protein abundance (Fig. 4C). Simultaneously, MKI treatment promoted the degradation of SQSTM1 (Fig. S6C), implying that exposure to MKIs led to autophagy activation. In line with this, MKI treatment also enhanced the interaction between YTHDF1 and GFP-LC3 (Fig. 4D).

It is well established that adaptor proteins mediate the degradation of autophagic cargoes [25]. Further screening of classic adaptor proteins revealed that the knockdown of *NBR1* and *OPTN* markedly reversed the MKI-induced decrease in YTHDF1 abundance (Fig. 4E and Fig. S6D). However, only the knockdown of *NBR1* but not *OPTN* reversed the MKI-induced decrease in DLAT aggregation (Fig. S6E) and specifically enhanced the cuproptosis induced by combined MKI and ES treatment (Fig. 4F to H and Fig. S6F to K). Accordingly, NBR1 was selected for further exploration of the mechanism by which MKIs promoted YTHDF1 down-regulation. We found that knocking down *NBR1* indeed reversed both the MKI-induced decline in YTHDF1 and FDX1 abundance, and protein lipoylation levels (Fig. 4I and Fig. S6L). Moreover, the combination of Sora and ES exhibited potent cytotoxicity and induced pronounced cuproptosis in HCC cells with stable *NBR1* knockdown in vivo (Fig. 4J to M). Combined, these results indicated that MKIs attenuated cuproptosis by triggering the NBR1-mediated autophagic degradation of YTHDF1.

### MKIs inhibited K515 methylation in YTHDF1, thereby promoting its association with NBR1

**Fig. 5.**
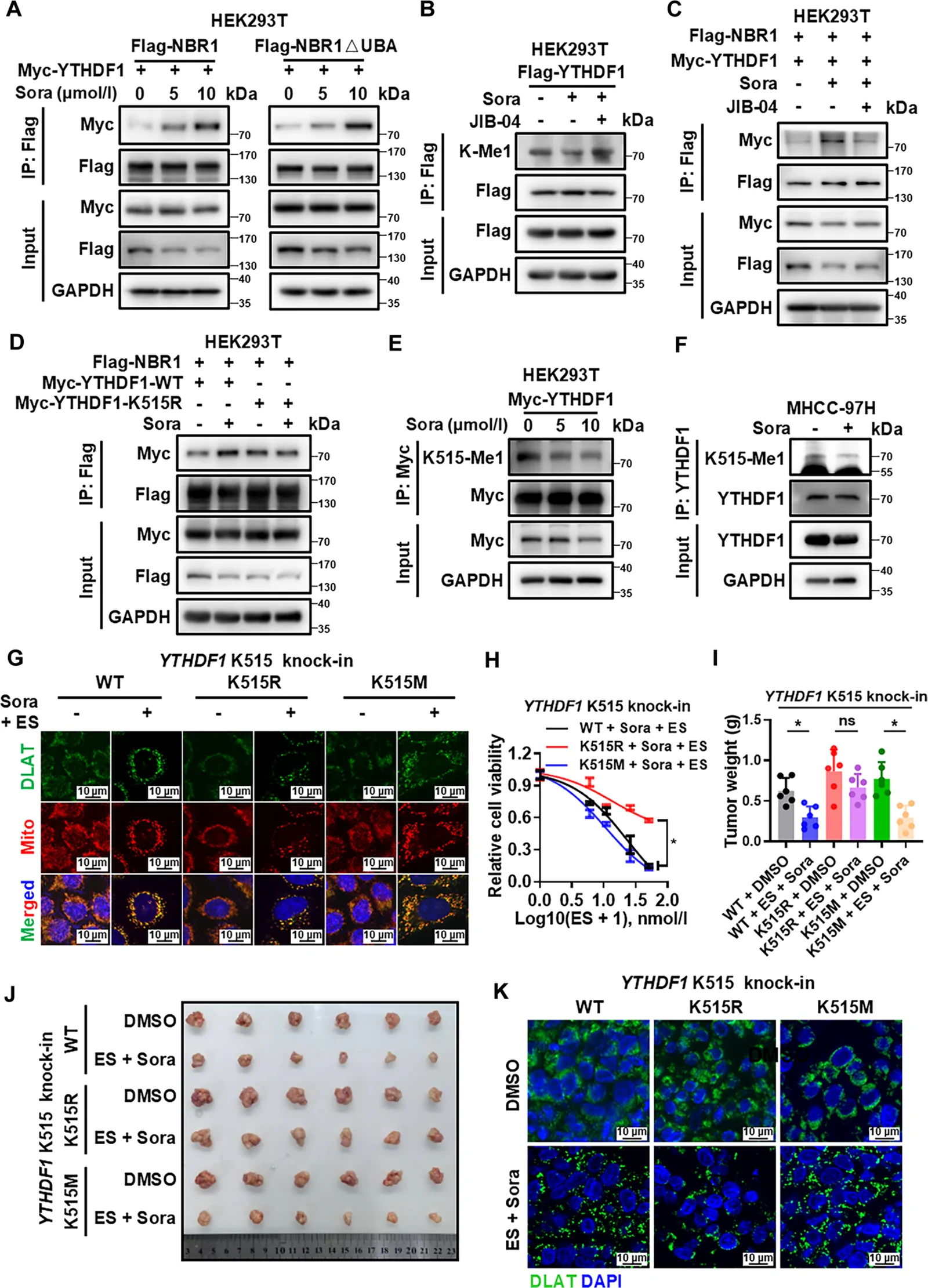
MKIs inhibited lysine 515 methylation in YTHDF1, thereby promoting its association with NBR1. (A) The interaction between Myc-YTHDF1 and Flag-NBR1 or Flag-NBR1ΔUBA under Sora treatment for 24 h was analyzed by co-IP and Western blotting in HEK293T cells. (B) The K-Me1 modification levels of Myc-YTHDF1 under 10 μmol/l Sora treatment for 24 h, together with or without 1 μmol/l JIB-04, were analyzed by IP and Western blotting in HEK293T cells. (C) The interaction between Myc-YTHDF1 and Flag-NBR1 under 10 μmol/l Sora treatment for 24 h with or without 1 μmol/l JIB-04 was analyzed by co-IP in HEK293T cells. (D) The interaction between Flag-NBR1 and Myc-YTHDF1-WT or Myc-YTHDF1-K515R under 10 μmol/l Sora treatment for 24 h was analyzed by co-IP in HEK293T cells. (E) K515-Me1 modification levels of Myc-YTHDF1 under indicated Sora treatment for 24 h were analyzed by IP in HEK293T cells. (F) K515-Me1 modification levels of endogenous YTHDF1 under 10 μmol/l Sora treatment for 24 h were analyzed by IP in MHCC-97H cells. (G) DLAT aggregation in *YTHDF1* K515 knock-in cells, comprising *YTHDF1*-WT, *YTHDF1*-K515R, and *YTHDF1*-K515M, under 25 nmol/l ES and 5 μmol/l Sora incubation for 48 h, controlled with DMSO, was analyzed by IF (green, DLAT; red, Mitotracker; blue, DAPI). (H) Cell viability of *YTHDF1* K515 knock-in cells, comprising *YTHDF1*-WT, *YTHDF1*-K515R, and *YTHDF1*-K515M, under incubation with ES and 5 μmol/l Sora for 48 h was measured by CCK-8. (I and J) Nude mice xenograft model (*n* = 6 per group) was generated by subcutaneous inoculation of *YTHDF1* K515 knock-in cells, comprising *YTHDF1*-WT, *YTHDF1*-K515R, and *YTHDF1*-K515M. Mice were then treated with indicated drugs comprising DMSO or ES + Sora 4 times a week. Tumor weight (I) and pictures (J) were summarized and shown respectively. (K) Tissue sections from each group of xenograft tumors were labeled with DLAT (green) and DAPI (blue) by IF. Abbreviations: MKIs, multikinase inhibitors; ΔUBA, UBA-deletion mutant of NBR1; Sora, sorafenib; co-IP, co-immunoprecipitation; K-Me1, lysine monomethylation; IP, immunoprecipitation; WT, wild type; K515R, lysine 515 mutated to arginine; K515M, lysine 515 mutated to methionine; K515-Me1, YTHDF1 K515 monomethylation; ES, elesclomol; CCK-8, Cell Counting Kit-8; IF, immunofluorescence; ns, not statistically significant.

Typically, NBR1 depends on its ubiquitin-associated (UBA) domain to recruit ubiquitinated substrates to the autophagosome for degradation [25]. However, the binding between YTHDF1 and NBR1 was still elevated after MKI treatment, even in the presence of NBR1ΔUBA (Fig. 5A and Fig. S7A). Moreover, MKIs did not alter the poly-ubiquitination of YTHDF1 (Fig. S7B). This indicated that ubiquitination modification did not mediate the MKI-induced enhancement of the interaction between YTHDF1 and NBR1. Thus, we screened other common posttranslational modifications, comprising methylation, acetylation, and phosphorylation, and found that MKI treatment decreased K-Me1 and increased K-Ace, but did not influence p-Tyr or p-Ser/Thr (Fig. S7C). Additionally, exposure to NAM or TSA, 2 broad-spectrum deacetylase inhibitors [26], did not down-regulate YTHDF1 protein levels (Fig. S7D). Conversely, treatment with the broad-spectrum demethylating inhibitor JIB-04 [27] effectively reversed the MKI-induced down-regulation of YTHDF1 (Fig. S7E). Furthermore, JIB-04 also reversed the decrease in K-Me1 levels and the enhanced interaction between YTHDF1 and NBR1 resulting from MKI treatment (Fig. 5B and C). These data revealed that MKIs promoted the NBR1-mediated autophagic degradation of YTHDF1 by inhibiting its K-Me1 modification.

Mass spectrometric analysis revealed that YTHDF1 might be monomethylated at multiple lysine sites comprising K371, K419, K515, and K541 [28]. By introducing the corresponding methylation-inactive mutations (lysine mutated to arginine, K>R), we confirmed that compared to the YTHDF1-WT, the YTHDF1-K515R mutant exhibited the greatest decrease in K-Me1 levels and the strongest promotive effect on its interaction with NBR1 (Fig. S8A and B). Importantly, unlike with YTHDF1-WT, MKI could not promote the interaction between YTHDF1-K515R and NBR1 (Fig. 5D). Domain mapping of YTHDF1 indicated that the region comprising amino acids 283 to 539 was responsible for NBR1 binding (Fig. S8C). In addition, YTHDF1 protein sequence analysis showed that the K515 site was conserved among vertebrates (Fig. S8D). Molecular docking prediction for YTHDF1 and NBR1 also indicated that residue K515 of YTHDF1 might play an important role in their interaction (Fig. S8E). Using a custom anti-K515-Me1 antibody, which recognized YTHDF1-WT but not YTHDF1-K515R or YTHDF1-K515M (Fig. S8F), we found that MKI treatment decreased the K515-Me1 levels in both exogenous and endogenous YTHDF1 (Fig. 5E and F). K515-Me1 modification levels in YTHDF1 were also substantially decreased in Sora-resistant HCC cells (Fig. S8G). In the context of ES combined with MKIs, overexpression of the YTHDF1-K515M mutant conferred the strongest sensitization effect, followed by YTHDF1-WT, while the YTHDF1-K515R mutant showed the weakest effect (Fig. S8H and I). Taken together, these data demonstrated that MKIs inhibited the K515-Me1 modification in YTHDF1, thereby promoting its recognition by NBR1 and its autophagic degradation.

To better validate the physiological role of YTHDF1 K515-Me1, CRISPR-Cas9 technology was employed to generate *YTHDF1*-K515R and *YTHDF1*-K515M knock-in mutations in HCC cells (Fig. S9A). Consistent with the above results, substitution of K515 with arginine rendered HCC cells tolerant to DLAT aggregation and cuproptosis induction (Fig. 5G and H and Fig. S9B and C). Meanwhile, the interaction between YTHDF1-K515R and NBR1 was markedly enhanced relative to that observed between YTHDF1-WT and NBR1 (Fig. S9D), and the half-life of YTHDF1-K515R was notably shorter than that of both YTHDF1-WT and YTHDF1-K515M (Fig. S9E). In cells expressing *YTHDF1*-K515R, the FDX1 protein levels were also substantially down-regulated (Fig. S9F). Finally, the tumor-suppressive effect of Sora and ES was markedly inferior in *YTHDF1*-K515R knock-in xenografts than in *YTHDF1*-WT or *YTHDF1*-K515M xenografts (Fig. 5I and J and Fig. S9G). This was closely associated with the inability of YTHDF1-K515R to induce robust cuproptosis (Fig. 5K). Overall, these data implied that MKIs inhibited the K515-Me1 modification in YTHDF1, thereby promoting NBR1-mediated autophagic degradation and ultimately inducing cuproptosis tolerance.

### MKIs inhibited the K515-Me1 modification in YTHDF1 by disrupting AKT-mTOR-SMYD2 signaling

We next sought to identify the specific methyltransferases and demethylases responsible for the K515-Me1 modification in YTHDF1. For this, we screened a siRNA library containing methyltransferases and demethylases for enzymes that influenced exogenous Myc-YTHDF1 protein levels and the interaction between YTHDF1 and NBR1. The screening identified JMJD7 as the potential YTHDF1 demethylase (Fig. S10A and B). The knockdown of *JMJD7* reversed MKI-induced suppression of both Myc-YTHDF1 protein levels (Fig. S10C) and its K515-Me1 modification (Fig. S10D). Moreover, *JMJD7* knockdown disrupted the MKI-induced enhancement of YTHDF1–NBR1 binding (Fig. S10E). In contrast, the application of exogenous *JMJD7* effectively reduced the abundance of the YTHDF1 K515-Me1 modification (Fig. S10F) and promoted the interaction between YTHDF1 and NBR1 (Fig. S10G). Notably, the knockdown of *JMJD7* reversed the cuproptosis tolerance induced by MKI pretreatment (Fig. S10H). These results confirmed that JMJD7 was the enzyme responsible for the demethylation of YTHDF1. *JMJD7* depletion increased lysine methylation in YTHDF1 and its subsequent stabilization, while its overexpression facilitated YTHDF1 degradation, leading to the suppression of *FDX1* translation. However, MKI failed to increase the association between JMJD7 and YTHDF1 (Fig. S10I), indicating that the MKI-induced decrease in K515-Me1 levels may result from changes in the activity of the relevant methyltransferase.

**Fig. 6.**
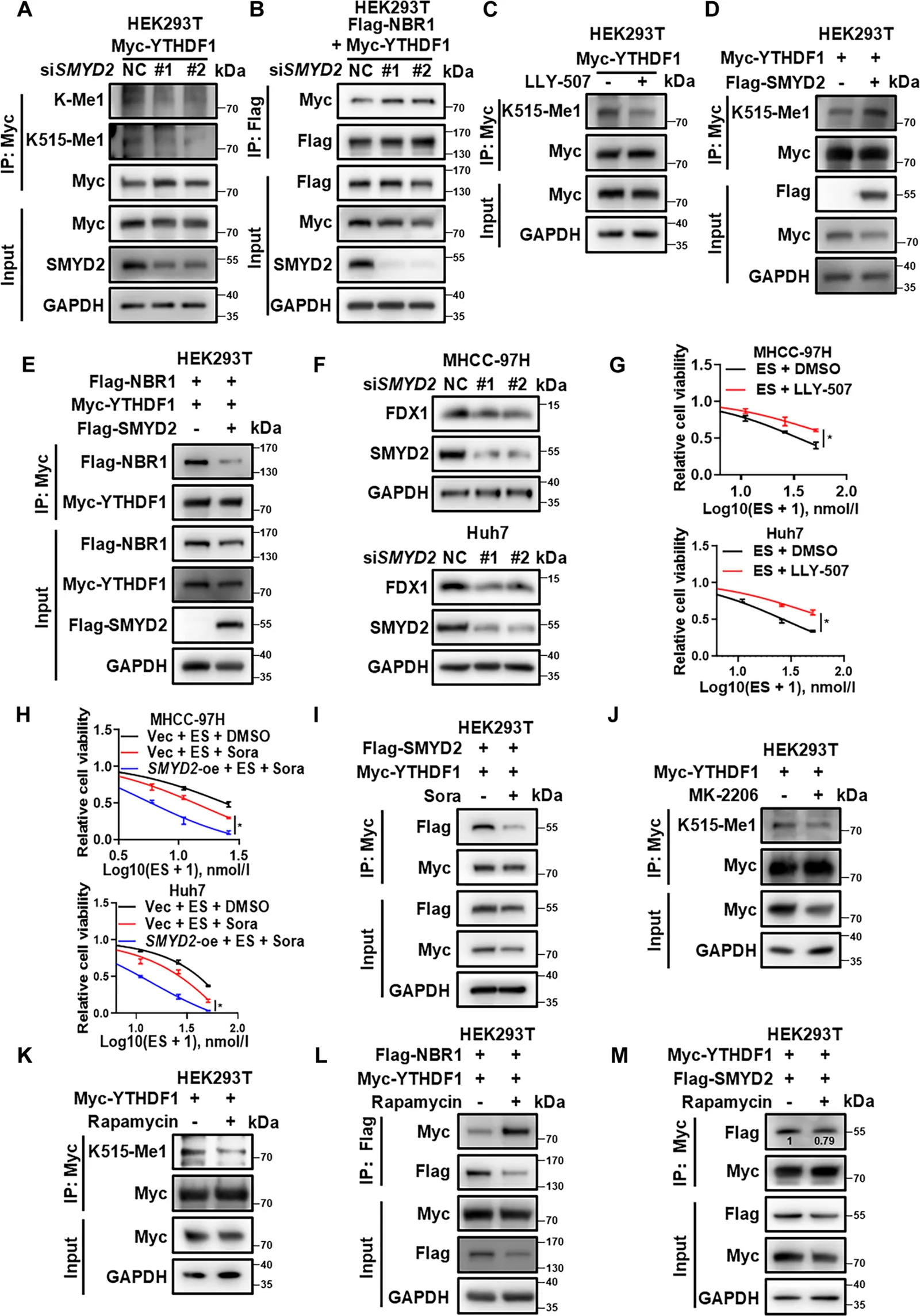
MKIs inhibited the K515-Me1 modification in YTHDF1 by disrupting AKT-mTOR-SMYD2 signaling. (A) The K-Me1 and K515-Me1 modification levels of Myc-YTHDF1 under *SMYD2* knockdown were analyzed by IP in HEK293T cells. (B) The interaction between Myc-YTHDF1 and Flag-NBR1 with or without *SMYD2* knockdown was analyzed by co-IP in HEK293T cells. (C) K515-Me1 modification levels of Myc-YTHDF1 under 5 μmol/l LLY-507 treatment for 48 h were analyzed by IP in HEK293T cells. (D) K515-Me1 modification levels of Myc-YTHDF1 under *SMYD2* overexpression were analyzed by IP in HEK293T cells. (E) The interaction between Myc-YTHDF1 and Flag-NBR1 with or without *SMYD2* overexpression was analyzed by co-IP in HEK293T cells. (F) FDX1 protein levels under *SMYD2* knockdown were analyzed by Western blotting in MHCC-97H and Huh7 cells. (G) Cell viability of MHCC-97H and Huh7 cells under ES with or without 5 μmol/l LLY-507 for 48 h was detected by CCK-8. (H) MHCC-97H and Huh7 cells overexpressing vector or *SMYD2* were treated with 5 μmol/l Sora and ES, and changes in cell viability were measured by CCK-8 assay. (I) The interaction between Myc-YTHDF1 and Flag-SMYD2 with or without 10 μmol/l Sora treatment for 24 h was analyzed by co-IP in HEK293T cells. (J and K) K515-Me1 modification levels of Myc-YTHDF1 under 10 μmol/l MK-2206 (J) or 20 μmol/l Rapamycin (K) treatment for 48 h were analyzed by IP in HEK293T cells. (L) The interaction between Myc-YTHDF1 and Flag-NBR1 under 20 μmol/l Rapamycin treatment for 48 h was analyzed by co-IP in HEK293T cells. (M) The interaction between Myc-YTHDF1 and Flag-SMYD2 with or without 20 μmol/l Rapamycin treatment for 48 h was analyzed by co-IP in HEK293T cells. Abbreviations: MKIs, multikinase inhibitors; K-Me1, lysine monomethylation; K515-Me1, YTHDF1 K515 monomethylation; IP, immunoprecipitation; co-IP, co-immunoprecipitation; ES, elesclomol; CCK-8, Cell Counting Kit-8; Vec, vector; Sora, sorafenib; *SMYD2*-oe, *SMYD2* overexpression.

Similarly, SMYD2 was identified as the methyltransferase potentially responsible for YTHDF1 K515 methylation (Fig. S11A to C). *SMYD2* knockdown effectively lowered the level of YTHDF1 K515-Me1 (Fig. 6A) and promoted the interaction between YTHDF1 and NBR1 (Fig. 6B). Treatment with LLY-507, an inhibitor of SMYD2, also reduced the level of YTHDF1 K515-Me1 (Fig. 6C). Conversely, *SMYD2* overexpression exerted the opposite effects (Fig. 6D and E). Additionally, the knockdown of *SMYD2* down-regulated the protein levels of FDX1 (Fig. 6F). Meanwhile, the pharmacological inhibition of SMYD2 abrogated sensitivity to cuproptosis (Fig. 6G), whereas its overexpression enhanced the cuproptosis-inducing efficacy of combined copper ionophore with MKI treatment (Fig. 6H). Furthermore, SMYD2-YTHDF1 binding was substantially weakened after MKI treatment (Fig. 6I and Fig. S11D). These observations implied that MKIs inhibited the K515-Me1 modification in YTHDF1 by disrupting the interaction between SMYD2 and YTHDF1.

As MKIs mainly inhibit the activity of RTKs such as VEGFR, PDGFR, FLT3, and c-Kit [29,30], we employed specific inhibitors of these 4 RTKs to determine which pathway regulated YTHDF1 methylation. We found that all 4 inhibitors reduced YTHDF1 protein levels in HCC cells (Fig. S11E). Therefore, we further screened downstream signaling pathways common to these RTKs (e.g., AKT-mTOR and MAPK) using the AKT inhibitor MK-2206, the mTOR inhibitor Rapamycin, and the mitogen-activated protein kinase kinase 1/2 inhibitor PD318088. After determining effective inhibitor concentrations (Fig. S11F and G), subsequent experiments revealed that the inhibition of either AKT or mTOR, but not MAPK signaling, markedly reduced YTHDF1 K515-Me1 modification levels (Fig. 6J and K and Fig. S11H). Meanwhile, the administration of either Rapamycin or MK-2206 substantially promoted the interaction between YTHDF1 and NBR1 (Fig. 6L and Fig. S11I) and reduced YTHDF1 protein levels (Fig. S11J). Rapamycin also inhibited the interaction between SMYD2 and YTHDF1 (Fig. 6M). Collectively, these results suggested that MKIs inhibited AKT-mTOR signaling, thereby suppressing the interaction between SMYD2 and YTHDF1 and reducing K515-Me1 modification levels in YTHDF1, ultimately promoting its autophagic degradation.

### SCDlip, a liposomal nanoplatform for the codelivery of multiple therapeutics, sensitized HCC cells to both MKI treatment and cuproptosis induction

**Fig. 7.**
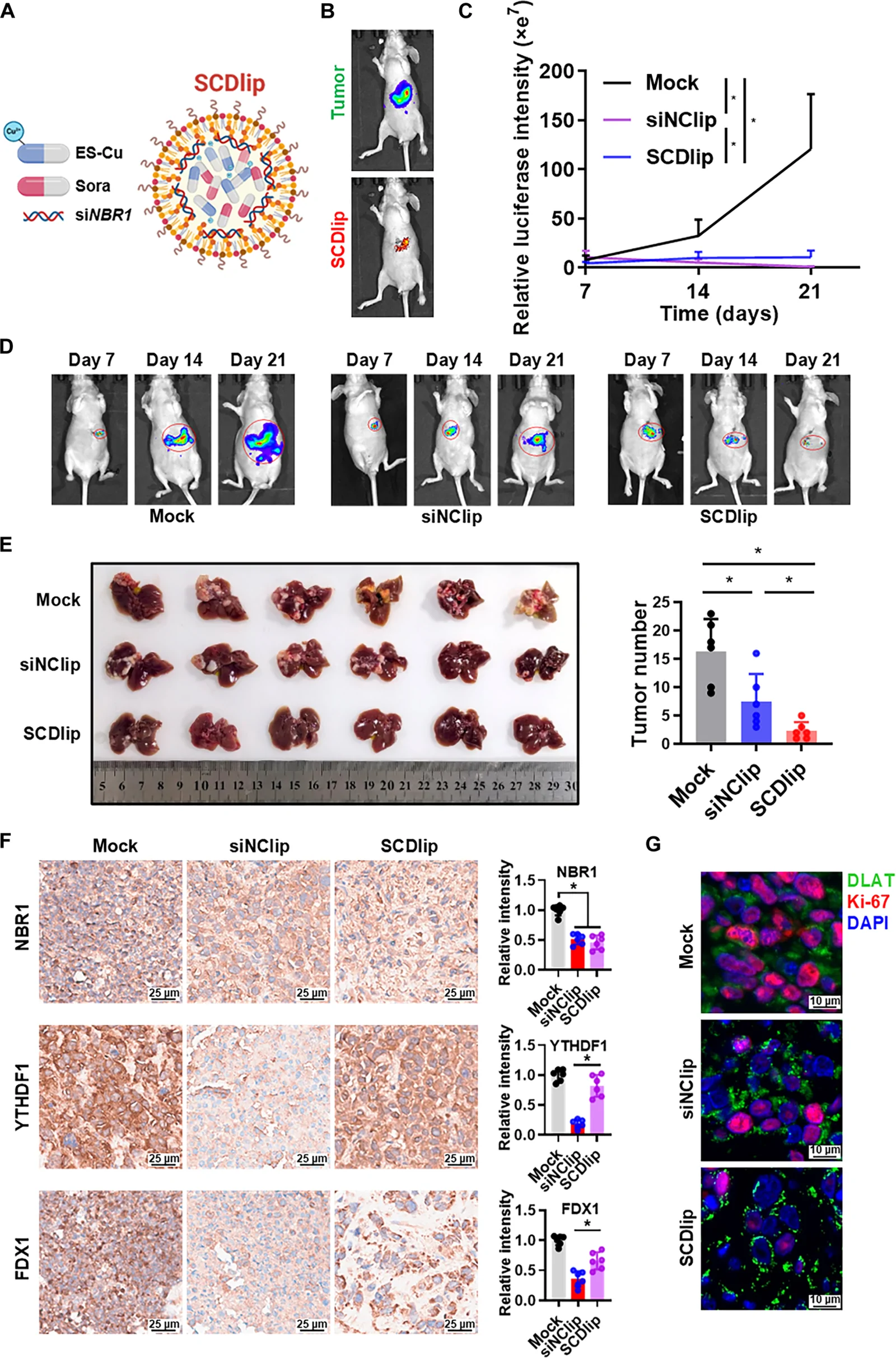
SCDlip, a liposomal nanoplatform for the codelivery of multiple therapeutics, sensitized HCC cells to both MKI treatment and cuproptosis induction. (A) Schematic diagram of SCDlip. The diagram was created from BioRender.com. (B) In vivo distribution of SCDlip in MHCC-97H luciferase-overexpression xenograft mouse was captured by small animal imaging. The top image showed the signal from tumor cells with luciferase, while the bottom image displayed the signal from Cy5-labeled SCDlip. (C to E) An in situ xenograft tumor model was established by implanting MHCC-97H luciferase-overexpression cells (*n* = 6 per group) in the liver. After administering the corresponding drugs, the luciferase signal intensity of the in situ tumors was monitored using a small animal imaging system. The relative luciferase intensity (C), representative luciferase pictures (D), and tumor pictures (E) were summarized and shown respectively. (F) Each group of xenograft tumor sections was stained with NBR1, YTHDF1, and FDX1 by IHC. (G) Tissue sections from each group of xenograft tumors were labeled with DLAT (green), Ki-67 (red), and DAPI (blue). Abbreviations: SCDlip, super cuproptosis detonator liposome; HCC, hepatocellular carcinoma; ES-Cu, Cu^2+^-elesclomol; IHC, immunohistochemistry.

The above findings implied that MKIs promoted the NBR1-mediated autophagic degradation of YTHDF1, thereby delaying *FDX1* translation and rendering HCC cells tolerance to both MKI treatment and cuproptosis induction. Thus, we hypothesized that blocking the autophagic degradation of YTHDF1 by targeting *NBR1* may improve the efficacy of MKI treatment and promote cuproptosis induction. To validate this possibility, we developed a liposomal nanoplatform—SCDlip—optimized for the codelivery of siRNA targeting *NBR1* (si*NBR1*), Sora, and ES-Cu (Fig. 7A). In a murine orthotopic in situ liver tumor model, Cy5-labeled SCDlip showed strong colocalization with orthotopic liver tumors (Fig. 7B), while in a subcutaneous xenograft model, Cy5-labeled SCDlip mainly accumulated in grafted tumor and the liver (Fig. S12A). This demonstrated that SCDlip effectively targeted tumor and the liver. Following SCDlip treatment, no notable abnormalities were detected in major organs, alleviating concerns regarding unacceptable toxicity (Fig. S12B). Regarding antitumor efficacy, liposomes coloaded with Sora, ES-Cu, and control siRNA (siNClip) exhibited moderate antitumor effects compared with the mock group, whereas SCDlip displayed a markedly superior antitumor effect compared with that observed with siNClip (Fig. 7C to E and Fig. S12C to E). Immunohistochemistry (IHC) analysis indicated that SCDlip, but not siNClip, effectively reversed the suppression of the YTHDF1–FDX1 axis (Fig. 7F). Moreover, SCDlip induced robust cuproptosis (increased DLAT aggregation) and inhibited the proliferation of HCC cells (decreased Ki-67 staining) in vivo (Fig. 7G). Combined, these findings suggested that *NBR1* inhibition restored FDX1 expression, which was essential for potentiating the efficacy of SCDlip through overcoming MKI resistance and promoting cuproptosis induction.

## Discussion

**Fig. 8.**
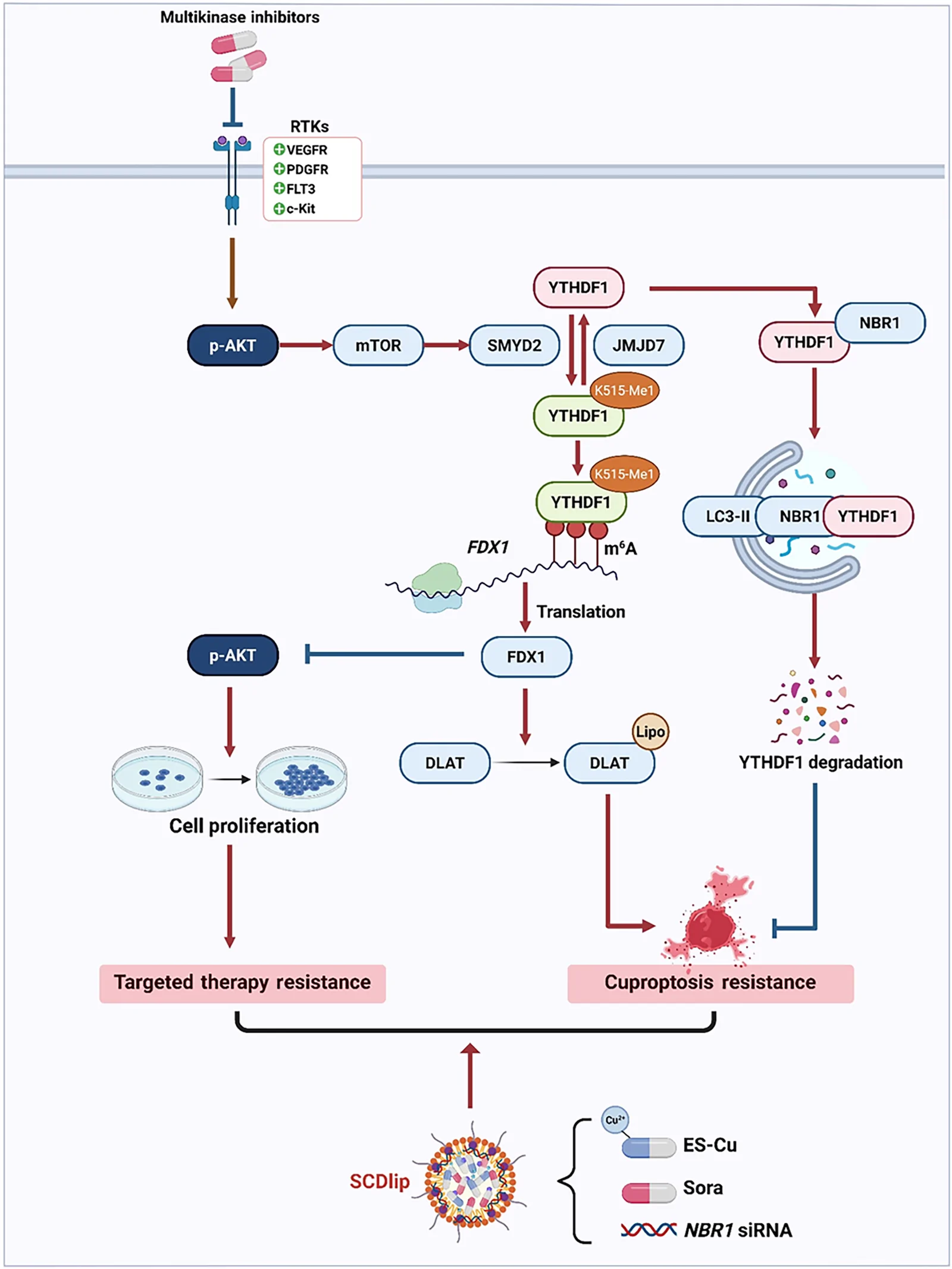
Working model. MKIs sorafenib and lenvatinib could inhibit AKT-mTOR signaling pathway to suppress the interaction between SMYD2 and YTHDF1, leading to the down-regulation of YTHDF1 K515-Me1, which activated NBR1-mediated YTHDF1 autophagic degradation. The decrease of YTHDF1 resulted in the inhibition of *FDX1* mRNA m6A-dependent translation, followed by protein lipoylation down-regulation, ultimately compromising cuproptosis in HCC. Furthermore, *FDX1* translation inhibition activated the AKT signaling pathway, which subsequently promoted resistance to MKIs. Using SCDlip targeting *NBR1* to restore FDX1 expression was a potential therapeutic strategy that could greatly enhance cuproptosis and the efficacy of targeted therapy in HCC. Arrows represented activating relationships between adjacent molecules, while T-shaped arrows denoted inhibition. The diagram was created from BioRender.com. Abbreviations: MKIs, multikinase inhibitors; K515-Me1, YTHDF1 K515 monomethylation; HCC, hepatocellular carcinoma; SCDlip, super cuproptosis detonator liposome.

The emergence of resistance to targeted therapies such as Sora represents a critical barrier in HCC management [31], necessitating a deeper understanding of the molecular adaptations that enable tumor survival. In this study, we identified the SMYD2–YTHDF1–FDX1 axis as a regulator of both cuproptosis sensitivity and MKI efficacy, thereby establishing FDX1 as a dual-function, metabolic–epigenetic checkpoint in HCC treatment. Our findings revealed how therapeutic pressure reshaped the tumor’s epitranscriptomic and metabolic landscape to evade both drug toxicity and copper-dependent cell death, while providing actionable strategies for counteracting these adaptations (Fig. 8).

We demonstrated that MKIs suppressed AKT-mTOR signaling, thereby abrogating YTHDF1 K515 methylation mediated by the methyltransferase SMYD2. This hypomethylation enabled the NBR1-mediated autophagic degradation of YTHDF1, impairing its role in *FDX1* mRNA translation. Thus, this finding linked epitranscriptomic regulation (m6A dynamics), proteostasis (autophagic turnover), and mitochondrial metabolism (FDX1-dependent lipoylation). Importantly, the loss of FDX1 was found to drive 2 parallel resistance mechanisms: reactivation of AKT survival signaling, thus sustaining tumor growth under MKI pressure; and failure to reduce Cu^2+^ to the more toxic Cu^+^ and sustain protein lipoylation levels, thereby promoting tolerance to copper overload. This dual role of FDX1 underscored its centrality as a metabolic gatekeeper whose down-regulation created a permissive environment for HCC adaptation. It means that restoring FDX1 expression achieves the dual therapeutic outcome of simultaneously reversing MKI resistance and reactivating cuproptosis. Indeed, accumulating evidence indicated that alterations in FDX1 expression or function influenced susceptibility to cuproptosis via the adjustment of protein lipoylation levels. In gastric cancer cells, the lactylation of METTL16 at lysine 229 was reported to enhance *FDX1* mRNA stability by increasing its m6A modification level, which substantially increased protein lipoylation and subsequently sensitized cancer cells to cuproptosis both in vitro and in vivo [32]. ARID1A deficiency in some cancer cells suppressed glycolysis, which in turn enhanced the tricarboxylic acid (TCA) cycle, and ultimately sensitized the cells to cuproptosis in an FDX1-dependent manner [33]. Consistent with previous reports [34], we confirmed that MKI treatment induced a metabolic shift toward glycolysis in HCC cells and further discovered that *FDX1* knockdown produced a similar effect (data not shown). Studies have shown that the lipoylation of mitochondrial proteins such as DLAT and DLST is important for the activity of the pyruvate dehydrogenase complex, whose E2 subunit is DLAT, and the α-ketoglutarate dehydrogenase complex, whose E2 subunit is DLST [35,36]. This suggests that reduced protein lipoylation might contribute to the inactivation of the TCA cycle and the activation of glycolysis. Moreover, enhanced glycolysis was reported to inhibit protein lipoylation in an FDX1-dependent manner, thus slowing the TCA cycle [33]. This intricate regulation suggests that *FDX1* overexpression following MKI treatment would restore protein lipoylation and concomitantly enhance the mitochondrial TCA cycle. Consequently, the metabolic shift toward glycolysis may be a reasonable indicator of cuproptosis tolerance.

Our work expanded the functional repertoire of m6A readers. We showed that YTHDF1 activity depended not only on its RNA-binding capacity but also on posttranslational modifications that protected YTHDF1 from NBR1-dependent autophagic degradation. Substrate recognition specificity in selective autophagy is primarily determined by the adaptor proteins including NBR1, SQSTM1, OPTN, TAX1BP1, and NDP52 [25]. These proteins typically recognize polyubiquitinated substrates and recruit them into the autophagosome [25,37]. No other posttranslational modifications other than ubiquitination have been identified as being necessary for substrate recognition by adaptor proteins. Here, we found that K515-Me1 prevented YTHDF1 from being recognized by the autophagy adaptor protein NBR1. MKI treatment impaired the interaction between YTHDF1 and its lysine methyltransferase SMYD2, thus enabling the NBR1-dependent autophagic degradation of YTHDF1. Further work is needed to determine whether other posttranslational modifications are involved in substrate recognition for autophagic degradation.

The finding that NBR1 acted as a selective autophagy receptor for hypomethylated YTHDF1 established this interaction as a druggable axis for HCC therapy. Pharmacologically stabilizing YTHDF1 via SMYD2 activation, JMJD7 inhibition or NBR1 inhibition may lead to the restoration of FDX1 levels, offering dual benefits: overcoming MKI resistance and reinstating susceptibility to cuproptosis. Numerous studies have recently highlighted the potential of nanoliposome platforms for the codelivery of nucleic acids targeting previously “undruggable” proteins. A notable example involved the cationic liposomes engineered to incorporate si*HIF1α* and Sora for the treatment of Sora-resistant HCC [38]. Our engineered SCDlip platform validated this approach, combining Sora and copper ionophores with *NBR1* depletion to bypass FDX1 suppression and achieving superior liver tumor targeting efficacy by leveraging the enhanced permeability and retention (EPR) effect [39,40]. The efficacy of SCDlip in vivo might be achieved by overcoming MKI resistance and reactivate cuproptosis, highlighting its translational potential, particularly for tumors with inherent or acquired SMYD2–YTHDF1–FDX1 axis defects. Notably, the SCDlip design addressed a key limitation of copper-based therapies—the inability to sustain lethal Cu^+^ accumulation in cancer cells—by up-regulating FDX1 expression via the targeting of NBR1.

The relevance of cuproptosis, a recently identified form of copper-linked cell death, to therapy resistance in solid tumors remains poorly defined. Our work indicated that cuproptosis evasion represented an adaptive mechanism co-opted by HCC cells under MKI pressure, akin to how apoptosis or ferroptosis resistance drove tolerance to other therapies [41,42]. For instance, Sora was shown to induce ferroptosis resistance through mechanisms involving metal metabolism regulation and epigenetic modifications. Specifically, Sora was found to increase 5-methylcytosine modification levels in metastasis-associated lung adenocarcinoma transcript 1, which promoted the cytoplasmic translocation of ELAV like RNA binding protein 1. This, in turn, enhanced solute carrier family 7 member 11 mRNA stability and up-regulated its expression, thereby suppressing ferroptosis and contributing to drug resistance [43]. Furthermore, Sora was demonstrated to activate the nuclear factor erythroid 2-related factor 2 transcription factor, leading to the up-regulation of metallothionein-1G expression, consequently inhibiting ferroptosis and leading to Sora resistance [44]. However, unlike these pathways, we found that MKIs had an unexpected dual role in modulating cuproptosis in HCC cells. On the one hand, MKIs promoted cuproptosis in the short-term by enhancing FDX1 protein stability and inhibiting glutathione synthesis [9]. On the other hand, under sustained exposure, MKIs suppressed *FDX1* translation, thereby blocking its de novo synthesis and ultimately inhibiting cuproptosis. Therefore, in MKI-resistant HCC (such as Sora resistance), consistently reduced FDX1 protein and protein lipoylation levels also contributed to cuproptosis resistance, as FDX1-dependent protein lipoylation was essential but not sufficient to activate cuproptosis until copper overload [9]. Consequently, targeting NBR1 not only counteracted the MKI-induced inhibition of *FDX1* translation but also leveraged the MKI beneficial effect of inhibiting mitochondrial hydrolase-dependent FDX1 protein degradation, thereby achieving a multipronged sensitization effect.

Following the establishment of the SMYD2–YTHDF1–FDX1 axis in preclinical models, clinical validation is needed to assess its relevance across HCC subtypes and etiologies (e.g., viral vs. nonviral). Mechanistically, future work could explore the contribution of changes in the mitochondrial membrane potential to cuproptosis induction. Efforts should also focus on optimizing the pharmacokinetics and safety profile of SCDlip while identifying biomarkers (e.g., YTHDF1 methylation status and FDX1 protein level) to determine which patients are most likely to benefit from cuproptosis-directed therapies. With the development of mRNA technology, it would also be plausible to package mRNAs encoding *YTHDF1*-K515M or *FDX1* in the nanoliposome as an alternative to *NBR1* knockdown, which may cause off-target effects. Nevertheless, this study focused exclusively on HCC, primarily because the liver is the major copper storage organ and is responsible for maintaining copper homeostasis [9,11,45]. Whether this potential strategy could be extended to other cancers where various therapeutics are employed to target AKT-mTOR signaling merits further exploration.

## Conclusions

In summary, we identified a hierarchical signaling axis centered on the SMYD2–YTHDF1–FDX1 cascade that governed how survival and cuproptosis were dynamically balanced in HCC under MKI stress. By coupling epitranscriptomic control of translation (YTHDF1) with mitochondrial metabolic rewiring (FDX1), tumors co-opted a single pathway to evade the effects of both MKIs and copper toxicity. Our findings delineated cuproptosis as a therapeutically actionable pathway and provided a blueprint for dual-targeting strategies aimed at disrupting resistance mechanisms in HCC.

## Ethical Approval

This study was approved by the animal ethics committee of Sir Run Run Shaw Hospital, Zhejiang University School of Medicine (No. SRRSH2023-0002). Animal care and experiments were conducted in compliance with Institutional Animal Care and Use Committee and National Institutes of Health guidelines.

## Data Availability

Proteomic data can be accessed from the National Genomics Data Center (OMIX012821).
