# Supplementary material for: NBR1-Mediated Autophagic Degradation of YTHDF1 Curtails FDX1 Translation to Drive Concurrent Multikinase Inhibitor Resistance and Cuproptosis Tolerance
Source: Cancer Commun (Lond). 2026 Sep 11;46:0048. doi: 10.34133/cancomm.0048 (PMC13562797; doi:10.34133/cancomm.0048)
Supplement: Supplementary file 1 [file cancomm.0048.f1.zip › Figs. S1 to S12 R1.docx]

**Supplementary Figures and Legends**

**Figure S1. FDX1 downregulation conferred MKIs resistance in HCC.** (A) The time course effect of 10 μmol/l Lenva on FDX1 protein levels were analyzed by Western blotting in MHCC-97H and Huh7 cells. (B) After *FDX1* knockdown, cell viability of MHCC-97H and Huh7 cells under Lenva treatment for 48 h was measured with CCK-8 assay.

Abbreviation: MKIs, multikinase inhibitors; HCC, hepatocellular carcinoma; Lenva, lenvatinib.

**Figure S2. MKIs decreased FDX1 to compromise cuproptosis induction.** (A and B) The levels of Lip-DLAT and Lip-DLST in MHCC-97H and Huh7 cells under dose gradient of 24 h Sora or Lenva treatment were analyzed by Western blotting. (C) After 10 μmol/l Lenva pretreatment for 48 h, controlled with DMSO, cell viability of MHCC-97H and Huh7 cells under ES and Lenva treatment for 48 h was measured with CCK-8 assay. (D) After 10 μmol/l Lenva pretreatment for 48 h in MHCC-97H and Huh7 cells, 25 nmol/l ES was added for 48 h. DLAT aggregation was analyzed by soluble-insoluble fraction isolation. (E) After 5 μmol/l Sora or 10 μmol/l Lenva pretreatment for 48 h in Huh7 cells, 25 nmol/l ES was added for 48 h. DLAT aggregation was analyzed by IF (green, DLAT; red, Mitotracker; blue, DAPI). (F) Western blotting analysis of the expression of lipoylated proteins (Lip-DLAT and Lip-DLST) with or without *FDX1* overexpression after 10 μmol/l Lenva 48 h treatment in MHCC-97H and Huh7 cells. (G) MHCC-97H and Huh7 cells were transfected with Myc-FDX1, controlled with empty vector, then pretreated with 10 μmol/l Lenva for 48 h, and DMSO was used as a control. After that, ES (50 nmol/l, controlled with DMSO) was further added 48 h, cell viability was measured by CCK-8 assay.

Abbreviation: MKIs, multikinase inhibitors; Lip-DLAT, lipoylated DLAT; Lip-DLST, lipoylated DLST; Lenva, lenvatinib; pre, pretreatment; ES, elesclomol; IF, immunofluorescence; Vec, vector; *FDX1*-oe, overexpression of *FDX1*.

**Figure S3. MKIs retarded *FDX1* translation in a YTHDF1-dependent manner.** (A) MHCC-97H cells with si*AFG3L2* and si*LONP1* co-transfection were treated with 5 μmol/l Sora for 48 h, DMSO was used as control. The protein levels of FDX1 were determined by Western blotting. (B) *FDX1* mRNA levels under Lenva 48 h treatment in MHCC-97H cells were analyzed by RT-qPCR. (C) Myc-FDX1 overexpressed MHCC-97H cells were incubated with 10 μmol/l Lenva for 48 h, and Western blotting was performed to detect the protein levels of Myc-FDX1. (D) After 5 μmol/l Sora (left) or 10 μmol/l Lenva (right) treatment for 48 h in MHCC-97H cells, 100 µg/ml CHX was added as the indicated times, and FDX1 protein turnover was analyzed by Western blotting. (E) Relative amount of *FDX1* mRNA in various polysome fractions under 5 μmol/l Sora or 10 μmol/l Lenva for 48 h was analyzed by RT-qPCR in Huh7 cells. Fractions 1–4 corresponded to the 40S and 60S ribosomal subunits, fractions 5–8 represented the monosome fraction, and fractions 9–13 indicated the polysome fraction. (F) Huh7 cells were co-transfected with *FDX1*-SunTag and scFv-GFP reporter plasmids, then 5 μmol/l Sora or 10 μmol/l Lenva was added for 48 h, controlled with DMSO. Newly synthesized FDX1 peptide signals were detected by IF. (G) *FDX1* mRNA m6A modification distribution was visualized by Integrative Genomics Viewer using the sequencing data of GSE149510. (H) Western blotting analysis of FDX1 protein levels after *METTL16* knockdown in MHCC-97H and Huh7 cells. (I) m6A levels of *FDX1* mRNA after *METTL16* knockdown in MHCC-97H and Huh7 cells were detected by MeRIP. (J to O) Western blotting analysis of FDX1 protein levels after *ALKBH5* (J), *IMP1* (K), *IMP2* (L), *IMP3* (M), *YTHDF3* (N) or *YTHDC2* (O) knockdown in MHCC-97H and Huh7 cells. (P) After 2 μmol/l Tega treatment for 24 h in MHCC-97H and Huh7 cells, FDX1 protein levels were analyzed by Western blotting. (Q) Binding of YTHDF1 to *FDX1* mRNA after *METTL3* knockdown in MHCC-97H and Huh7 cells was detected by RIP.

Abbreviation: MKIs, multikinase inhibitors; Sora, sorafenib; Lenva, lenvatinib; RT-qPCR, reverse transcription quantitative real-time PCR; CHX, cycloheximide; IF, immunofluorescence; MeRIP, methylated RNA immunoprecipitation; Tega, tegaserod; RIP, RNA immunoprecipitation.

**Figure S4. m6A modification regulated the translation of *FDX1* mRNA.** (A) Relative amount of *FDX1* mRNA in various polysome fractions after *YTHDF1* knockdown was analyzed by RT-qPCR in Huh7 cells. Fractions 1–4 corresponded to the 40S and 60S ribosomal subunits, fractions 5–8 represented the monosome fraction, and fractions 9–13 indicated the polysome fraction. (B) Relative amount of *FDX1* mRNA in various polysome fractions after *METTL3* knockdown was analyzed by RT-qPCR in MHCC-97H and Huh7 cells. Fractions 1–4 corresponded to the 40S and 60S ribosomal subunits, fractions 5–8 represented the monosome fraction, and fractions 9–13 indicated the polysome fraction. (C) After *YTHDF1* knockdown, *FDX1* SunTag reporter system was introduced into Huh7 cells. And newly synthesized FDX1 peptide signals were detected by IF. (D) After *METTL3* knockdown, *FDX1* SunTag reporter system was introduced into MHCC-97H and Huh7 cells. And newly synthesized FDX1 peptide signals were detected by IF. (E) After Sora or Lenva treatment for 48 h in MHCC-97H and Huh7 cells, *FDX1* mRNA m6A levels were detected by MeRIP. (F) After 10 μmol/l Lenva treatment for 48 h in MHCC-97H and Huh7 cells, YTHDF1, FTO and METTL3 protein levels were analyzed by Western blotting. (G) The effect of Flag-YTHDF1 overexpression on FDX1 protein levels under 10 μmol/l Lenva treatment for 48 h was analyzed by Western blotting in MHCC-97H and Huh7 cells. (H) MHCC-97H and Huh7 cells were transfected with Flag-YTHDF1/vector, then pretreated with 5 μmol/l Sora (in Huh7 cells) or 10 μmol/l Lenva (in both cells) for 48 h. After that, ES was added for another 48 h, and cell viability was measured by CCK-8 assay.

Abbreviation: RT-qPCR, reverse transcription quantitative real-time PCR; IF, immunofluorescence; Sora, sorafenib; Lenva, lenvatinib; MeRIP, methylated RNA immunoprecipitation; pre, pretreatment; ES, elesclomol.

**Figure S5. Suppression of YTHDF1 reduced cuproptosis susceptibility in HCC *in vitro* and *in vivo*.** (A) After *YTHDF1* knockdown in Huh7 cells, ES was added for 48 h, and cell viability was measured with CCK-8 assay. (B) After *YTHDF1* knockdown in MHCC-97H and Huh7 cells, DDC was added for 48 h, and cell viability was measured with CCK-8 assay. (C) MHCC-97H cells were transfected with Myc-FDX1, together with si*YTHDF1*, controlled with empty vector or siNC. Then 50 nmol/l ES was added for 48 h, DMSO was used as a control, and cell viability was measured with CCK-8 assay. (D) Cell viability of MHCC-97H or Huh7 cells with ES and 2 μmol/l Tega 48 h treatment was measured with CCK-8 assay. (E) MHCC-97H and Huh7 cells were transfected with si*YTHDF1* or siNC, and 25 nmol/l ES was added for 48 h. DLAT aggregation was analyzed by soluble-insoluble fraction isolation. (F) MHCC-97H and Huh7 cells were treated with 2 μmol/l Tega and 25 nmol/l ES for 48 h, and DLAT aggregation was analyzed by soluble-insoluble fraction isolation. (G) Huh7 cells were transfected with si*YTHDF1* or siNC, and 25 nmol/l ES was added for 48 h, DLAT aggregation was analyzed by IF (green, DLAT; red, Mitotracker; blue, DAPI). (H) MHCC-97H and Huh7 cells were treated with 2 μmol/l Tega and 25 nmol/l ES for 48 h, and DLAT aggregation was analyzed by IF (green, DLAT; red, Mitotracker; blue, DAPI). (I to K) Nude mice xenograft model (*n* = 6 per group) was generated by subcutaneous inoculation of MHCC-97H cells. Mice were then treated with indicated drugs comprising DMSO, ES, Tega, ES + Tega four times a week. Tumor pictures (I), growth curve (J) and weight (K) were summarized and shown respectively. (L) Tumor tissue sections from each group of MHCC-97H xenograft model were labeled with DLAT (green) and DAPI (blue) by IF.

Abbreviation: HCC, hepatocellular carcinoma; ES, elesclomol; DDC, ditiocarb sodium; CCK-8, Cell Counting Kit-8; Tega, tegaserod; IF, immunofluorescence; ns, not statistically significant.

**Figure S6. MKIs triggered NBR1-mediated autophagic degradation of YTHDF1.** (A) After Sora or Lenva treatment for 48 h in MHCC-97H cells, *YTHDF1* mRNA levels were analyzed by RT-qPCR. (B) Flag-YTHDF1 overexpressed MHCC-97H cells were treated with Sora for 48 h, and Flag-YTHDF1 protein levels were analyzed by Western blotting. (C) After Sora or Lenva treated for 12 h in MHCC-97H cells, YTHDF1 and SQSTM1 protein levels were analyzed by Western blotting. (D) After MHCC-97H or Huh7 cells were transfected with siRNA of indicated autophagy adaptors respectively, 10 μmol/l Lenva was added for 48 h, controlled with DMSO. The protein levels of YTHDF1 were analyzed by Western blotting. (E) *NBR1* or *OPTN* knockdown MHCC-97H cells were pretreated with 5 μmol/l Sora for 48 h, then 25 nmol/l ES was added for another 48 h. DLAT aggregation was analyzed by soluble-insoluble fraction isolation. (F) *OPTN* knockdown MHCC-97H or Huh7 cells were co-treated with 5 μmol/l Sora and ES together for 48 h, controlled with DMSO, and cell viability was measured by CCK-8. (G) After *NBR1* or *OPTN* knockdown in MHCC-97H and Huh7 cells, 10 μmol/l Lenva was added for 48 h, together with ES, and cell viability was measured with CCK-8. (H) *NBR1* knockdown MHCC-97H and Huh7 cells were treated with 25 nmol/l ES for 48 h, together with 10 μmol/l Lenva, and controlled with DMSO. DLAT aggregation was analyzed by soluble-insoluble fraction isolation. (I) *OPTN* knockdown MHCC-97H and Huh7 cells were treated with 25 nmol/l ES for 48 h, together with 5 μmol/l Sora or 10 μmol/l Lenva, and controlled with DMSO. DLAT aggregation was analyzed by soluble-insoluble fraction isolation. (J) *NBR1* knockdown Huh7 cells were co-treated with 25 nmol/l ES and 5 μmol/l Sora for 48 h, controlled with DMSO, and DLAT aggregation was analyzed by IF (green, DLAT; red, Mitotracker; blue, DAPI). (K) *OPTN* knockdown MHCC-97H or Huh7 cells were co-treated with 25 nmol/l ES and 5 μmol/l Sora for 48 h, controlled with DMSO, and DLAT aggregation was analyzed by IF (green, DLAT; red, Mitotracker; blue, DAPI). (L) *NBR1* knockdown MHCC-97H and Huh7 cells were treated with 10 μmol/l Lenva for 48 h, and the levels of YTHDF1, FDX1, Lip-DLAT and Lip-DLST were analyzed by Western blotting.

Abbreviation: MKIs, multikinase inhibitors; Sora, sorafenib; Lenva, lenvatinib; RT-qPCR, reverse transcription quantitative real-time PCR; pre, pretreatment; ES, elesclomol; CCK-8, Cell Counting Kit-8; IF, immunofluorescence; Lip-DLAT, lipoylated DLAT; Lip-DLST, lipoylated DLST; ns, not statistically significant.

**Figure S7. MKIs promoted the association of YTHDF1 with NBR1 via inhibiting its lysine monomethylation.** (A) The interaction between Myc-YTHDF1 and Flag-NBR1 or Flag-NBR1△UBA under 10 μmol/l Lenva treatment for 24 h in HEK293T cells was analyzed by co-IP. (B) Myc-YTHDF1 overexpressed HEK293T cells were treated with 10 μmol/l Sora or 10 μmol/l Lenva for 24 h, poly-ubiquitin of YTHDF1 was determined by IP. (C) Flag-YTHDF1 overexpressed HEK293T cells were treated with 10 μmol/l Sora or DMSO for 24 h, and the canonical protein post-translational modifications such as K-Me1, p-Tyr, p-Ser/Thr and K-Ace were determined by IP. (D) The protein levels of YTHDF1 after 1 mmol/l NAM or 0.1 μmol/l TSA 24 h treatment in MHCC-97H were analyzed by Western blotting. (E) MHCC-97H cells were incubated with 10 μmol/l Sora for 24 h, together with or without 1 μmol/l JIB-04, the YTHDF1 protein levels were analyzed by Western blotting.

Abbreviation: MKIs, multikinase inhibitors; △UBA, NBR1 lacking the UBA domain; Lenva, lenvatinib; co-IP, co-immunoprecipitation; Sora, sorafenib; IP, immunoprecipitation; K-Me1, lysine monomethylation; p-Tyr, tyrosine phosphorylation; p-Ser/Thr, serine/threonine phosphorylation; K-Ace, lysine acetylation; NAM, nicotinamide; TSA, trichostatin A.

**Figure S8. MKIs promoted the association of YTHDF1 with NBR1 via inhibiting its lysine 515 methylation. (**A) The K-Me1 modification levels of Myc-YTHDF1-WT and mutants in HEK293T cells were analyzed by IP. (B) The interaction between Flag-NBR1 and Myc-YTHDF1-WT or mutants in HEK293T cells was analyzed by co-IP. (C) The interaction between Myc-NBR1 and full length Flag-YTHDF1 or truncations in HEK293T cells was analyzed by co-IP. (D) The conservation of YTHDF1 K515 in vertebrates was shown. (E) Molecular docking of YTHDF1 and NBR1 was performed using HDOCK server and visualized by PyMOL. (F) YTHDF1 K515-Me1 modification levels of Myc-YTHDF1-WT, Myc-YTHDF1-K515R and Myc-YTHDF1-K515M mutants were analyzed by IP in HEK293T cells. (G) K515-Me1 modification levels of endogenous YTHDF1 in 97H-P and 97H-SR were analyzed by IP. (H and I) After MHCC-97H was transfected with Myc-YTHDF1-WT, Myc-YTHDF1-K515R, Myc-YTHDF1-K515M or empty vector, 5 μmol/l Sora and ES/DDC were added for 48 h, and cell viability was measured with CCK-8.

Abbreviation: MKIs, multikinase inhibitors; K-Me1, lysine monomethylation; WT, wild type; K515R, lysine 515 mutated to arginine; IP, immunoprecipitation; co-IP, co-immunoprecipitation; FL, full length; K515-Me1, YTHDF1 K515 monomethylation; K515M, lysine 515 mutated to methionine; 97H-P, parental MHCC-97H cells; 97H-SR, sorafenib resistant MHCC-97H cells; Sora, sorafenib; ES, elesclomol; DDC, ditiocarb sodium; CCK-8, Cell Counting Kit-8.

**Figure S9. Construction of *YTHDF1* K515 mutants knock-in cell lines and functional validation.** (A) Schematic diagram of *YTHDF1* K515 knock-in mutants’ construction with CRISPR-Cas9. The diagram was created from BioRender.com. (B) DLAT aggregation in *YTHDF1*-WT, *YTHDF1*-K515R and *YTHDF1*-K515M MHCC-97H knock-in cells with 5 μmol/l Sora and 25 nmol/l ES incubation for 48 h was analyzed by soluble-insoluble fraction isolation. (C) Cell viability of *YTHDF1*-WT, *YTHDF1*-K515R and *YTHDF1*-K515M MHCC-97H knock-in cells with 5 μmol/l Sora and DDC incubation for 48 h was measured by CCK-8. (D) The interaction between endogenous NBR1 and YTHDF1 in YTHDF1-WT, YTHDF1-K515R MHCC-97H knock-in cells was analyzed by co-IP. (E) The *YTHDF1*-WT, *YTHDF1*-K515R and *YTHDF1*-K515M MHCC-97H knock-in cells were incubated with 100 µg/ml CHX in indicated times, protein stability of endogenous YTHDF1 was analyzed by Western blotting. (F) Protein levels of FDX1 in *YTHDF1*-WT, *YTHDF1*-K515R and *YTHDF1*-K515M MHCC-97H knock-in cells were analyzed by Western blotting. (G) Tumor growth curve of mice xenograft model using *YTHDF1*-WT, *YTHDF1*-K515R and *YTHDF1*-K515M MHCC-97H knock-in cells under indicated treatment was shown.

Abbreviation: K515, lysine 515; WT, wild type; K515R, lysine 515 mutated to arginine; K515M, lysine 515 mutated to methionine; Sora, sorafenib; DDC, ditiocarb sodium; CCK-8, Cell Counting Kit-8; ES, elesclomol; co-IP, co-immunoprecipitation; CHX, cycloheximide; ns, not statistically significant.

**Figure S10. JMJD7 was responsible for the demethylation of YTHDF1 K515.** (A) After co-transfected of Myc-YTHDF1 and indicated siKDMs in MHCC-97H cells respectively, 5 μmol/l Sora was added for 48 h. The protein levels of Myc-YTHDF1 were analyzed by Western blotting. (B) Myc-NBR1 and Flag-YTHDF1 were co-transfected into HEK293T cells, together with siNC/si*KDM4E*/si*JMJD4*/si*JMJD7*/si*JMJD10* respectively, then 10 μmol/l Sora was added 24 h. co-IP was performed to determine the interaction between Myc-NBR1 and Flag-YTHDF1. (C) The *JMJD7* knockdown MHCC-97H cells were treated with or without 5 μmol/l Sora for 48 h, YTHDF1 protein levels were analyzed by Western blotting. (D) Myc-YTHDF1 and siNC/si*JMJD7* were co-transfected into HEK293T cells, then treated with 10 μmol/l Sora for 24 h. The K-Me1 and K515-Me1 modification levels of Myc-YTHDF1 were analyzed by IP. (E) Flag-NBR1 and Myc-YTHDF1 were co-transfected into HEK293T cells, together with siNC/si*JMJD7*, then treated with 10 μmol/l Sora for 24 h. The interaction between Myc-YTHDF1 and Flag-NBR1 was analyzed by co-IP. (F) Flag-YTHDF1 and Myc-JMJD7/vector were co-transfected into HEK293T cells, the K-Me1 and K515-Me1 modification levels of Flag-YTHDF1 were analyzed by IP. (G) The interaction between Myc-YTHDF1 and Flag-NBR1 with or without *JMJD7* overexpression in HEK293T cells was analyzed by co-IP. (H) The *JMJD7* knockdown MHCC-97H and Huh7 cells were pretreated with 5 μmol/l Sora for 48 h, controlled with DMSO, then ES was further added for 48 h. Cell viability was measured with CCK-8. (I) The interaction between Flag-YTHDF1 and Myc-JMJD7 with or without 10 μmol/l Sora treatment for 24 h was analyzed by co-IP in HEK293T cells.

Abbreviation: K515, lysine 515; siKDMs, siRNA targeting lysine demethylases; Sora, sorafenib; co-IP, co-immunoprecipitation; K-Me1, lysine monomethylation; K515-Me1, YTHDF1 K515 monomethylation; IP, immunoprecipitation; pre, pretreatment; ES, elesclomol; CCK-8, Cell Counting Kit-8.

**Figure S11. MKIs inhibited K515-Me1 of YTHDF1 by impairing AKT-mTOR-SMYD2 signaling.** (A and B) After co-transfected of Myc-YTHDF1 and indicated siKMTs in MHCC-97H cells, the Myc-YTHDF1 protein levels were analyzed by Western blotting. (C) Myc-YTHDF1 and Flag-NBR1 were co-transfected into HEK293T cells, together with siNC/si*SMYD2*/si*SETD7*/si*SMYD3* respectively, the interaction between Myc-YTHDF1 and Flag-NBR1 was analyzed by co-IP. (D) The interaction between Myc-YTHDF1 and Flag-SMYD2 with or without 10 μmol/l Lenva treatment for 24 h was analyzed by co-IP in HEK293T cells. (E) After MHCC-97H cells were incubated with inhibitors of different RTKs for 24 h, YTHDF1 protein levels were analyzed by Western blotting. (F) Cell viability of MHCC-97H cells under MK-2206, Rapamycin or PD318088 treatment was measure by CCK-8. (G) The effects of 10 μmol/l MK-2206, 20 μmol/l Rapamycin, and 100 μmol/l PD318088 on the AKT-mTOR, mTOR, and MAPK signaling pathways, respectively, were assessed by Western blotting in MHCC-97H cells. (H) K515-Me1 modification levels of Myc-YTHDF1 in HEK293T cells under 100 μmol/l PD318088 treatment for 48 h were analyzed by IP. (I) The interaction between Myc-YTHDF1 and Flag-NBR1 under 10 μmol/l MK-2206 treatment for 48 h was analyzed by co-IP in HEK293T cells. (J) YTHDF1 protein levels in MHCC-97H cells with 10 μmol/l MK-2206 or 20 μmol/l Rapamycin 48 h treatment were analyzed by Western blotting.

Abbreviation: MKIs, multikinase inhibitors; K515-Me1, YTHDF1 K515 monomethylation; siKMTs, siRNA targeting lysine methyltransferases; co-IP, co-immunoprecipitation; RTKs, receptor tyrosine kinases; CCK-8, Cell Counting Kit-8; IP, immunoprecipitation.

**Figure S12. SCDlip, a multi-therapeutics co-loaded liposome, sensitized HCC cells to both MKI treatment and cuproptosis induction.** (A) Cy5-labeled SCDlip (controlled with saline) was injected via tail vein into the subcutaneous tumor‑bearing mouse, and the distribution of SCDlip was visualized by a small animal imaging system after 5 min. Subsequently, the mice were euthanized, and the distribution of SCDlip in the heart, liver, spleen, lung, and subcutaneous tumor was further examined via small animal imaging after dissection. (B) H&E staining of multiple tissue sections comprising heart, liver, spleen, lung and kidney from mock or SCDlip treated mice were shown. (C to E) Nude mice xenograft model (*n =* 6 per group) was generated by subcutaneous inoculation of Huh7 cells. Mice were then treated with indicated liposomes. Tumor pictures (C), weight (D) and growth curve (E) were summarized and shown respectively.

Abbreviation: SCDlip, super cuproptosis detonator liposome; HCC, hepatocellular carcinoma; MKI, multikinase inhibitor; H&E, hematoxylin and eosin.
